# Essential Role of Triplet Diradical Character for Large Magnetoresistance in Quinoidal Organic Semiconductor with High Electron Mobility

**DOI:** 10.1002/advs.202201045

**Published:** 2022-03-28

**Authors:** Chao Wang, Hua Hao, Keisuke Tajima

**Affiliations:** ^1^ RIKEN Center for Emergent Matter Science (CEMS) 2‐1 Hirosawa Wako Saitama 351‐0198 Japan

**Keywords:** diradicaloid, field‐effect transistor, organic magnetoresistance, organic semiconductor, spintronics

## Abstract

A diradicaloid molecule with high semiconducting performance is synthesized based on the quinoidal benzo[1,2‐b:4,5‐b′]dithiophene structure. The diradical character is investigated by quantum chemical calculations and variable temperature electron spin resonance. The diode devices based on this molecule show a large change in electric current in magnetic fields below 100 mT with a strong dependence on the measurement temperatures; as the population of the triplet diradicals increases at high temperatures, the magnetoconductance (MC) values increase. As a result, a MC of −19.4% is achieved at 120 °C, which is the largest negative MC observed for organic molecules to date. In contrast, a smaller diradicaloid molecule based on quinoidal thieno[3,2‐b]thiophene without thermally accessible triplet state shows no MC, indicating the essential role of the triplet diradicals. The strong correlation between the MC and the triplet diradical concentrations suggests that the charge conduction in the diradicaloid is suppressed through a spin‐blocking mechanism, which can be controlled through the magnetic modulation of the hyperfine fields. The compound forms high‐crystallinity thin films and has high monopolar electron transport in organic field‐effect transistors, with an average mobility of 1.01 cm^2^ V^−1^ s^−1^ for edge‐cast films.

## Graphical abstract

The diode device based on a novel diradicaloid molecule (DTBDTCN) exhibits the largest negative magnetoconductance (−19.4%) for organic molecules to date, which has unprecedented correlation with the concentration of the triplet diradicals. DTBDTCN also shows the high electron mobility of 1.01 cm^2^ V^−1^ s^−1^ in organic field effect transistor.

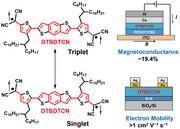

## 1 Introduction

Developments of various organic semiconductors with high performance have led to the attractive applications of solid‐state optoelectronic devices such as organic field effect transistor (OFET), organic light emitting diode, and organic photovoltaics. Adding spin freedom of the charge carriers into the working principle of the devices makes an emerging field of organic spintronics, which can lead to new applications such as magnetic memory devices with high density and fast operation speed.^[^ ^1^ ^]^ Organic semiconductors generally have small spin‐orbital coupling to preserve the spin states and thus the ability of long spin coherent charge transport. This unique property enables to construct bistable magnetic memory devices called spin valves by using the organic semiconductor layers sandwiched with two magnetic electrodes.^[^ ^2^ ^]^ On the other hand, spin filter effects of chiral organic molecules could lead to the selective injection of up/down spin and transport in the organic devices which is also applicable to the magnetic memory devices.^[^ ^3^ ^]^ Although the developments of the theory and the device fabrications are steadily progressing, organic spintronics is still at its infancy from the viewpoint of the materials chemistry and much effort on the synthesis of the new materials specifically oriented to the application in spintronics is highly anticipated.

Organic magnetoresistance (OMAR), in which a small magnetic field (<100 mT) can change the electric current, is observed in organic semiconductors.^[^ ^4^, ^5^, ^6^, ^7^, ^8^, ^9^ ^]^ Unlike the spin valves or the spin filters, OMAR can be observed in devices with no magnetic electrodes, and thus the phenomenon originates from the spin properties intrinsic to the organic semiconductors. OMAR is interesting not only from the viewpoint of physics but also from its potential applications, for example, in magnetoresistive sensors.^[^ ^10^ ^]^ Many common organic semiconductors have been investigated as OMAR device active layers, including tris(8‐hydroxyquinoline)aluminum (Alq_3_), poly(9,9‐di‐n‐octylfluorenyl‐2,7‐diyl) (PFO), 4,4,4‐tris[3‐methylphenyl(phenyl)amino]triphenylamine (m‐MTDATA), tris‐[3‐(3‐pyridyl)mesityl]borane (3TPYMB), poly(3‐hexylthiophene) (P3HT), poly[2‐methoxy‐5‐(3’,7’‐dimethyloctyloxy)‐1,4‐phenylenevinylene] (MDMO‐PPV), phenyl‐C_61_‐butyric acid methyl ester (PCBM), poly[2‐methoxy‐5‐(2’‐ethylhexyloxy)‐1,4‐phenylenevinylene] (MEH‐PPV), poly(*N*‐vinyl carbazole) (PVK), and pentacene.^[^ ^11^, ^12^, ^13^, ^14^, ^15^, ^16^, ^17^, ^18^, ^19^, ^20^, ^21^, ^22^ ^]^

*π*‐Conjugated diradicaloid molecules are attracting interest because of their potentially unique electronic properties originating from their diradical character.^[^ ^23^, ^24^, ^25^, ^26^, ^27^, ^28^, ^29^ ^]^ Diradicaloids have resonance structures between open‐shell diradicals and closed‐shell quinoids, the stabilities of which depend strongly on the molecular structures. In the diradical form, intramolecular spin coupling can occur between two electrons to form either singlet or triplet states. The chemical stability of the diradicaloids under ambient conditions is generally low and molecular structures with high stability are being sought. Applications of diradicaloids in organic electronics, spintronics, and non‐linear optics have been proposed.^[^ ^30^, ^31^, ^32^, ^33^ ^]^ In OFETs, diradicaloids often show ambipolar conduction due to the low band gaps, although monopolar conduction has also been reported.^[^ ^34^, ^35^, ^36^, ^37^, ^38^ ^]^ The highest hole and electron mobilities reported for OFETs based on diradical semiconductors are 1.4^[^ ^35^ ^]^ and 0.32 cm^2^ V^−1^ s^−1^,^[^ ^38^ ^]^ respectively. The high mobility has been ascribed to the enhanced intermolecular interaction through the singlet states of diradicaloids. However, the electronic properties that are truly unique to diradicaloids have not been elucidated, considering that ambipolar transport and high charge mobility have also been observed in highly crystalline closed‐shell organic semiconductors with low band gaps. We hypothesize that the diradical characters of the organic semiconductors can modulate the charge transport properties in the magnetic fields through the electronic spin interactions with the charge carriers. However, to our knowledge, OMAR effects of the organic semiconductors with the diradical character have not been reported to date. It was recently shown that the magnetoresistance in graphene and MoS_2_ can be tuned by a diradicaloid molecule attached on the surface,^[^ ^39^ ^]^ but in this case the charge transport occurs in the 2D materials, not in the organic molecules.

**Figure 1:**
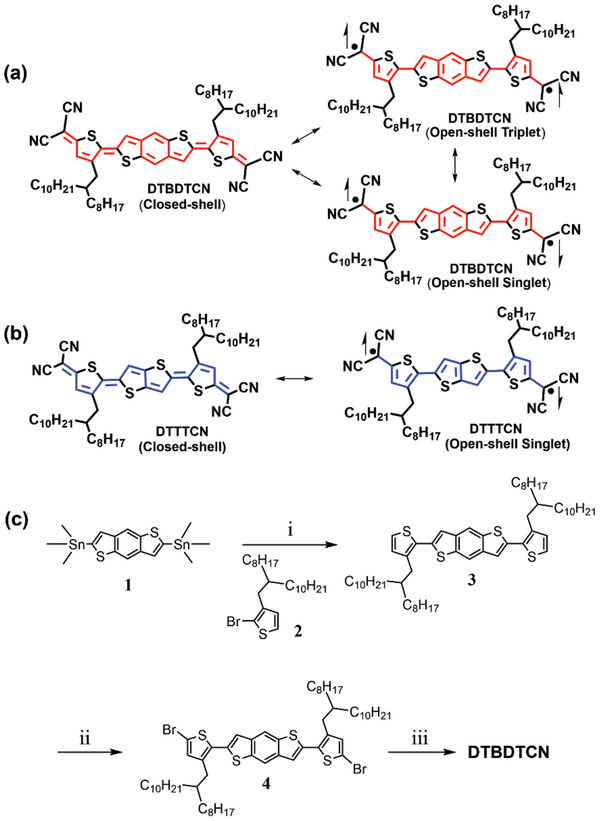
a) Closed‐shell, open‐shell singlet and triplet diradical forms of DTBDTCN. b) Closed‐shell and open‐shell singlet forms of DTTTCN. c) Synthetic routes for DTBDTCN. Reagents and conditions: i) Pd(PPh_3_)_4_, DMF, 120 °C, 10 h; ii) a) *n*‐BuLi, THF, −78 °C, 1 h, then CBr_4_, overnight; iii) malononitrile, NaH, Pd(PPh_3_)_4_, dimethoxyethane, reflux, 7 h, then HCl, DDQ, 2 h.

In this study, a quinoidal molecule based on dicyanomethylene‐substituted benzo[1,2‐b:4,5‐b′]dithiophene (DTBDTCN) was designed and synthesized toward the development of high‐performance semiconducting materials with diradical character. DTBDTCN has resonance structures among the closed‐shell quinoid and the open‐shell singlet and triplet diradicals (**Figure** **1**a). The molecular structure of DTBDTCN offers two advantages as an organic semiconductor. First, the strongly electron‐withdrawing dicyanomethylene groups at the ends stabilize the diradicals through conjugation effects and lower the lowest unoccupied molecular orbital (LUMO) levels to improve the device stability under ambient conditions. Second, the efficient intermolecular spin‐spin interaction of the diradical species together with the high planarity and rigidity of the quinoidal conjugated backbones can facilitate efficient charge transport.^[^ ^40^, ^41^ ^]^ While investigating the effect of the spin‐state on the charge conduction, we found a strong link between the triplet diradical characters and the OMAR effects in DTBDTCN. To further confirm this link, a diradicaloid molecule of dicyanomethylene‐substituted thieno[3,2‐b]thiophene (DTTTCN, Figure 1b) was also synthesized and used as a reference for OMAR and OFET measurements. Unlike DTBDTCN, DTTTCN does not have thermally accessible open‐shell triplet form as shown below.

## 2 Results and Discussion

### 2.1 Material Synthesis

The synthetic route for DTBDTCN is shown in Figure 1c. Compounds **1** and **2**, which were prepared by literature methods,^[^ ^42^, ^43^ ^]^ were subjected to palladium‐catalyzed Stille coupling to afford **3** in 78% yield. Treatment of **3** with *n*‐BuLi and CBr_4_ gave dibromide precursor **4** in 72% yield. Precursor **4** was subjected to palladium‐catalyzed coupling with sodium dicyanomethanide followed by oxidation with 2,3‐dichloro‐5,6‐dicyano‐1,4‐benzoquinone (DDQ) to afford DTBDTCN as a brown solid in 32% yield. DTTTCN was synthesized using a similar method as in the literature.^[^ ^44^ ^]^ The detailed synthetic procedures are described in the Supporting Information. The structures of the intermediates and the target molecule were fully characterized by nuclear magnetic resonance spectroscopy (NMR) and high‐resolution mass spectrometry (HRMS).

### 2.2 Quantum Chemical Calculations

The diradical character of DTBDTCN was first investigated by quantum chemical calculations based on density functional theory (DFT) at the UB3LYP/6‐31G(d,p) level of theory, and the results are summarized and compared with the other diradicaloids in the literatures in Table S1, Supporting Information. For the model structure of DTBDTCN with methyl groups for the side chains (Figure S1, Supporting Information), the structures were optimized with the restricted and unrestricted spin configurations. The optimized structures of the closed‐shell and open‐shell singlet showed the bond length alternation and the aromatic structures, respectively (Figure S2, Supporting Information), that were expected from the structures in Figure 1a. The most stable electronic configuration was the open‐shell singlet state. The open‐shell triplet state and the closed‐shell state were less stable than the open‐shell singlet by 0.114 eV (2.63 kcal mol^−1^) and by 0.147 eV (3.39 kcal mol^−1^), respectively (Table S1, Supporting Information). These results indicate that DTBDTCN had the open‐shell structure with the singlet state as the ground state, but the triplet state could be thermally populated with a small number of molecules at room temperature. A diradical character value (*y*_0_) of 0.594 was obtained for DTBDTCN by calculations according to a literature method.^[^ ^45^ ^]^ On the other hand, for the model structure of DTTTCN with methyl groups for the side chains (Figures S3 and S4, Supporting Information), the electronic energy of the closed‐shell state was only slightly higher that of the open‐shell singlet by 0.0065 eV (0.149 kcal mol^−1^, Table S1, Supporting Information), and the open‐shell triplet state was less stable than the open‐shell singlet by 0.278 eV (6.41 kcal mol^−1^). Therefore, the triplet states of DTTTCN are hardly thermally populated. The calculated *y*_0_ of DTTTCN is 0.186, much smaller than that of DTBDTCN, indicating DTTTCN possessed weaker diradical characters.

### 2.3 Variable‐Temperature ^1^H NMR

**Figure 2:**
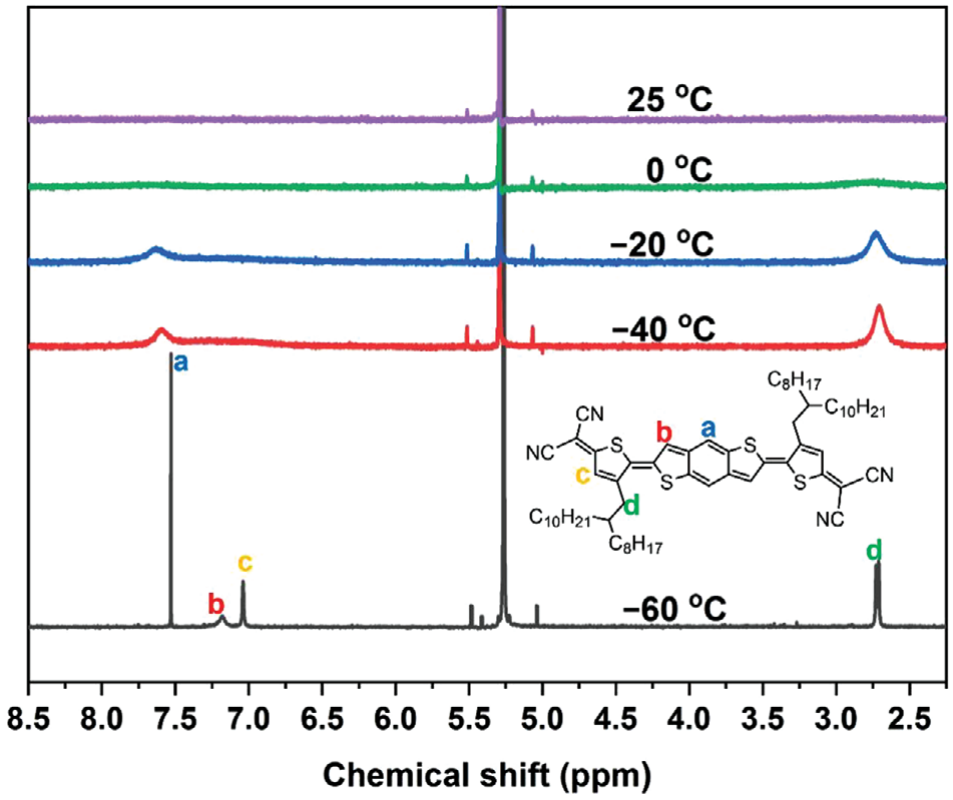
VT ^1^H NMR spectra of DTBDTCN in CD_2_Cl_2_ and assignment of the protons in the quinoidal *π*‐conjugated backbone and *α*‐methylene of the alkyl side chains.

The diradical character of DTBDTCN was suggested by variable‐temperature (VT) ^1^H NMR spectra (**Figure** **2**). The solution of DTBDTCN in CD_2_Cl_2_ showed no resonance signal for the protons on the quinoidal backbone or the *α*‐methylene of the alkyl side chains at 25 °C, whereas the other protons on the alkyl side chains appeared at 0.8–1.9 ppm (Figure S5, Supporting Information). This result suggests that the presence of the triplet state diradical interferes with NMR. Upon cooling, the peaks of both the quinoidal backbone and the *α*‐methylene began to appear with the temperature‐dependent sharpening. At −60 °C, the backbone proton peaks (a, b, and c in Figure 2) and *α*‐methylene peak of the alkyl groups (d) became sharp. This suggests that the diradical forms a singlet at the lower temperature and the interference was suppressed. Similar behaviors have been observed for diradicaloid molecules with triplet state energy slightly higher than that of the singlet state for the diradical,^[^ ^46^, ^47^ ^]^ suggesting a thermally populated triplet state. In contrast, DTTTCN showed sharp signals for the protons both on conjugated backbone and alkyl chains in the temperature range from −40 to 40 °C (Figures S6 and S42, Supporting Information), indicating the absence of the triplet diradical.

### 2.4 Electron Spin Resonance

**Figure 3:**
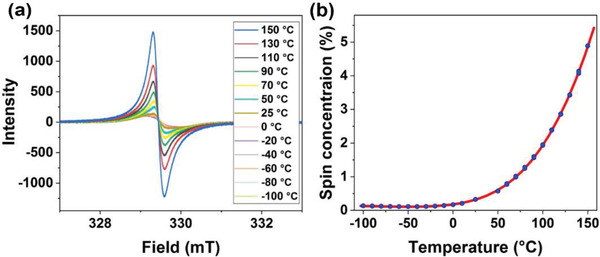
a) VT‐ESR spectra of DTBDTCN in the solid state. b) Spin concentration versus temperature (blue circles) and fitting with the Bleaney‐Bowers equation (red line).

To quantify the population of the triplet state in DTBDTCN and its dependence on the temperature, VT‐electron spin resonance (ESR) spectra were measured for DTBDTCN powder. A broad ESR signal with a *g*‐value of 2.004 was observed over the full temperature range (**Figure** **3**a). As the temperature was increased from −100 °C, the ESR signal intensity increased slightly up to 25 °C, whereas it increased strongly above 50 °C. The spin concentration in DTBDTCN was calculated from the double integrals of the ESR signals relative to the Mn standard that was calibrated with a standard solution sample of 4‐hydroxy‐2,2,6,6‐tetramethylpiperidin‐1‐oxyl (TEMPOL). The temperature dependence of the spin concentration, which is defined as the ratio between the number of the unpaired spins and the molecules, is shown in Figure 3b (blue circles). The spin concentration increased exponentially depending on the temperature, which suggested the thermally activated formation of the triplet. The fitting of the data with the Bleaney‐Bowers equation based on a singlet‐triplet model (red line) yielded a singlet‐triplet gap of 0.14 eV, which was consistent with the value from DFT calculations (0.114 eV). This result confirmed that DTBDTCN had diradical character with the singlet as the ground state and a thermally populated triplet state. In contrast, the powder of **DTTTCN** showed no signal in ESR at the temperatures in the range from 25 to 150 °C (Figure S7, Supporting Information), confirming the absence of the triplet diradicals according to the too high energy of the triplet state.

### 2.5 Thermal Properties

The thermal properties of DTBDTCN were investigated by thermogravimetric analysis (TGA) and differential scanning calorimetry (DSC). TGA showed that the thermolysis onset temperature for DTBDTCN was 398 °C, and the melting point obtained by DSC was 215 °C (Figure S8a,b, Supporting Information). The thermal analysis results suggested that the thermal stability of DTBDTCN was high, allowing the analysis of thin‐film crystallinity and morphology over a broad range of annealing temperatures. A large irreversible exothermic peak emerged on the DSC curve at 230 °C (Figure S8b, Supporting Information), whereas there was no obvious change in the TGA curve. We speculated that DTBDTCN diradicals may dimerize or polymerize in the liquid state, as previously observed for similar tetracyano quinoidal molecules^[^ ^48^ ^]^ (Figure S9, Supporting Information). The thermal properties of DTTTCN were different from those of DTBDTCN; TGA showed that DTTTCN powder started to decompose at 240 °C (Figure S8c, Supporting Information). In DSC, the melting point of DTTTCN also appeared at 240 °C and several exothermic peaks appeared at above this temperature (Figure S8d, Supporting Information), indicating DTTTCN could decompose in the liquid state.

### 2.6 Electronic Properties

**Figure 4:**
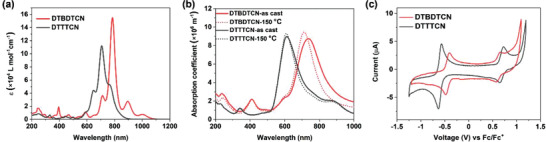
UV–vis–NIR spectra of DTBDTCN and DTTTCN in a) solution and in b) thin films on quartz substrates. c) CV of DTBDTCN and DTTTCN.

**Figure** **4**a,b shows the ultraviolet‐visible‐near‐IR (UV–vis–NIR) absorption spectra of materials in diluted CHCl_3_ solution and thin films. DTBDTCN showed a series of structured long‐wavelength absorption bands extending to 1100 nm in solution, with a maximum absorption peak at 783 nm (Figure 4a). In comparison with DTBDTCN, the absorption of DTTTCN in solution was in the shorter wavelength region with an absorption maximum at 707 nm. For the films of DTBDTCN, the absorption of the as‐cast film was blue shifted and broadened relative to the solution spectrum, with the absorption maximum at 736 nm (Figure 4b). Compared with the as‐cast film, the DTBDTCN film annealed at 150 °C showed a further blue shift in the absorption, with the absorption maximum at 711 nm and an increase in the absorption intensity, which could be caused by crystallization during annealing. Both the as‐cast film and the annealed film of DTBDTCN were blue. When the temperature was raised to 260 °C (above the melting point), the film of DTBDTCN turned pale yellow with a much lower absorption intensity, and the material became insoluble in organic solvents (Figure S10, Supporting Information). The color change of DTBDTCN film during thermal annealing at 260 °C agrees with the diradical polymerization suggested by DSC. For DTTTCN thin films, the absorption of the as‐cast film was also blue shifted and broadened compared to the solution spectrum with the absorption maximum at 609 nm (Figure 4b). However, the DTTTCN film absorption showed only slight change in wavelength and intensity after annealing at 150 °C, suggesting the effect of annealing on the molecular packing in DTTTCN film may not be large.

Cyclic voltammetry (CV) measurements were conducted to investigate the electrochemical properties and energy levels of DTBDTCN and DTTTCN. Both DTBDTCN and DTTTCN showed one reversible reduction and one reversible oxidation in the CV curve (Figure 4c). LUMO and the highest occupied molecular orbital (HOMO) energy levels of DTBDTCN calculated from the half‐wave potentials for the reduction and oxidation were −4.45 and −5.42 eV, respectively. Thus, the energy band gap was determined as 0.97 eV for DTBDTCN. For DTTTCN, the LUMO/HOMO energy levels were estimated to be −4.19 and −5.49 eV, respectively, indicating DTTTCN had a much larger energy gap (1.30 eV) than DTBDTCN.

The stabilities of DTBDTCN and DTTTCN were tested by monitoring the change in UV–vis–NIR absorbance at 781 and 707 nm in toluene solution under ambient conditions, respectively. The absorption half‐lives of the DTBDTCN and DTTTCN were estimated as 539 and 550 h, respectively (Figures S11 and S12, Supporting Information). The high stabilities can be ascribed to the deep HOMO energy levels of these two molecules which prevent the reactions with oxygen molecules.

### 2.7 OMAR Effects

**Figure 5:**
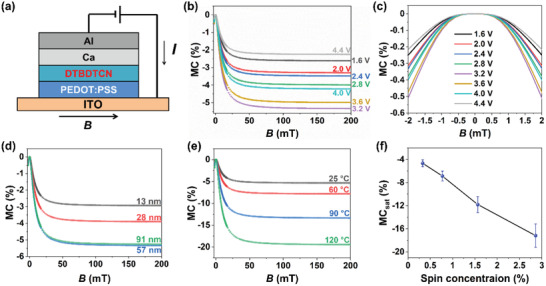
OMAR properties of DTBDTCN. a) Schematic of the device and the magnetoresistance experiment. b) MC versus *B* at different voltages. c) MC versus *B* at different voltages with low magnetic fields. d) MC versus *B* at 3.2 V with different DTBDTCN film thicknesses. e) MC versus *B* at 3.2 V at the different temperatures. f) MC_sat_ as a function of the spin concentration in DTBDTCN determined by ESR. The average MC_sat_ data were obtained from three devices.

Diode devices of DTBDTCN were fabricated with the structure indium tin oxide (ITO)/poly(3,4‐ethylenedioxythiophene) polystyrene sulfonate (PEDOT:PSS)/DTBDTCN/Ca/Al to investigate the OMAR effects of the diradicaloid semiconductor (**Figure** **5**a). Thickness of the spin‐coated DTBDTCN layer was about 60 nm unless otherwise noted. Magnetoconductance (MC) was used to quantify the OMAR effect, and is defined as MC = [*I*(*B*)−*I*(0)]/*I*(0), where *I*(B) and *I*(0) are the current at a constant voltage under magnetic field *B* and in the absence of a magnetic field, respectively. To avoid the effects of the thermally induced structural changes on MC, the DTBDTCN films for OMAR measurements were fully crystallized by annealing at 150 °C for 30 min before the electrode deposition, which would keep the morphology and crystallinity unchanged during the measurements at the various temperatures (see Thin Film Morphology Section in Supporting Information for the crystallization behaviors). The current–voltage (*I–V*) characteristics in Figure S13, Supporting Information shows rectifying behaviors. Considering the energy levels of the materials, the diode could be operating as a bipolar device (i.e., the hole injection at PEDOT:PSS and the electron injection at Ca interfaces). Figure 5b,c shows the MC of DTBDTCN films at room temperature (25 °C) as a function of the magnetic field with various applied voltages. MC was negative at all voltages and saturated under magnetic fields above 100 mT. The saturated MC (MC_sat_) was defined as the value at 200 mT and was plotted against the applied voltage (Figure S14a, Supporting Information). MC_sat_ reached an optimum value at 3.2 V of −5.3%, which is among the largest negative MC value observed for organic molecules at room temperature.

The thickness dependence of MC at room temperature is shown in Figure 5d and Figure S14b, Supporting Information. Negative MC was observed at all thicknesses (Figure 5d), and the absolute value of MC_sat_ increased as the DTBDTCN film thickness increased from 13 to 57 nm, and then slightly decreased at a film thickness of 91 nm (Figure S14b, Supporting Information). This dependence on the thickness suggests that the OMAR effect of DTBDTCN is originated from the bulk of the films rather than the electrodes/organic interfaces.

The temperature dependence of MC was measured from room temperature to 120 °C. At all temperatures, MC was negative, and the absolute value of MC_sat_ increased continuously with the temperature from room temperature to 120 °C (Figure 5e and Figure S14c, Supporting Information). This observation differs from most of the previous reports about common organic semiconductors in which the temperature dependence of OMAR effect was small.^[^ ^14^, ^21^ ^]^ MC_sat_ of the DTBDTCN device reached up to −19.4% at 120 °C, which is the largest negative MC observed for organic molecules to date. The change of the current was easily observed by using an ordinary current meter when a permanent magnet was moved closer to or away from the device (Video S1, Supporting Information, Figure S15, Supporting Information), demonstrating the potential application as a magnetoresistive sensor. After the measurements at 120 °C, when MC was measured again at a lower temperature, the devices showed almost the same trace as the first sweep at this lower temperature (Figure S16, Supporting Information). Therefore, the OMAR effect was reversible in the temperature range up to 120 °C. Figure 5f shows the plot of MC_sat_ as a function of the spin concentration (defined as the double of the triplet diradical concentration) determined by ESR. As the thermally populated open‐shell triplet state in DTBDTCN increased at the high temperatures, the saturated MC values become larger with an almost linear relationship with the concentrations of triplet diradicals. This strong correlation between the OMAR effect and the triplet diradical concentration in DTBDTCN suggests that the triplet diradicals play an important role in the electric conduction modulated by the magnetic field.

To further confirm that the triplet state played important role for MC, the diode device was also fabricated with DTTTCN that has the diradical character but much higher energy for the triplet state. DTTTCN device (ITO/PEDOT:PSS/DTTTCN/Ca/Al) showed no detectable OMAR effect (Figure S22, Supporting Information) in the temperature range up to 120 °C. This result also suggests that the triplet diradical character of DTBDTCN was important for the large OMAR effect.

To investigate the charge polarity responsible for the OMAR effect, unipolar device structures based on DTBDTCN were also investigated. Electron‐only devices were fabricated with the structures of ITO/ethoxylated polyethyleneimine (PEIE)/DTBDTCN/Ag and ITO/PEIE/DTBDTCN/Ca/Al. In these devices, PEIE was employed as a thin surface modifier that lowers the work function of ITO.^[^ ^49^ ^]^ On the other hand, ITO/PEDOT:PSS/DTBDTCN/MoO_3_/Ag was used as an hole‐only device structure, in which both MoO_3_ and PEDOT:PSS were used as the hole injection layers. The *I–V* curves of all the unipolar devices are nearly symmetric to the origin (Figure S13a, Supporting Information), suggesting that the carrier injection rates from the anode and the cathode are approximately similar. The *I–V* characteristics of all devices exhibited no hysteresis during forward and reverse sweep of bias voltage with a constant magnetic field of 100 mT (Figure S13b, Supporting Information), which does not suggest polarizations or charge trapping effects. As shown in Figures S17 and S18, Supporting Information, the electron‐only devices (ITO/PEIE/DTBDTCN/Ag and ITO/PEIE/DTBDTCN/Ca/Al) displayed the negative MC at different voltages, and their largest MC_sat_ at room temperature were −1.4% and −2.1%, respectively. The MC of these two devices showed strong temperature dependence, just like that of the bipolar device (ITO/PEDOT:PSS/DTBDTCN/Ca/Al), and MC_sat_ of them reached up to −10.5% and −13.9%, respectively, at 120 °C (Figures S17d and S18d, Supporting Information). The thickness dependence of MC for ITO/PEIE/DTBDTCN/Ag at room temperature is shown in Figure S17e,f, Supporting Information. MC_sat_ increased as the DTBDTCN film thickness increased from 15 to 61 nm, and then became unchanged when the thickness was 95 nm. In addition, strong temperature‐dependent OMAR effect was also observed when the electron‐only devices were operated at reversed biases of −5.2 and −4.4 V (Figures S19 and S20, Supporting Information) when the electron injection was switched to the PEIE/ITO side. These results further support that the OMAR effect of DTBDTCN is a bulk effect in the semiconducting layer, rather than the effects at the organic/electrodes interfaces. The plot of MC_sat_ as a function of spin concentrations also reveals strong correlation between OMAR effect and triplet diradical concentration in the electron‐only device (Figures S17g, S18e, S19c, and S20c, Supporting Information). In striking contrast to the bipolar and the electron‐only devices, the hole‐only device showed no OMAR effect independent from the measurement temperatures and the bias voltages (Figure S21, Supporting Information). These results suggest that the OMAR effect of DTBDTCN device could be attributed to the electron transport, not to the hole transport or the hole–electron recombination processes.

**Figure 6:**
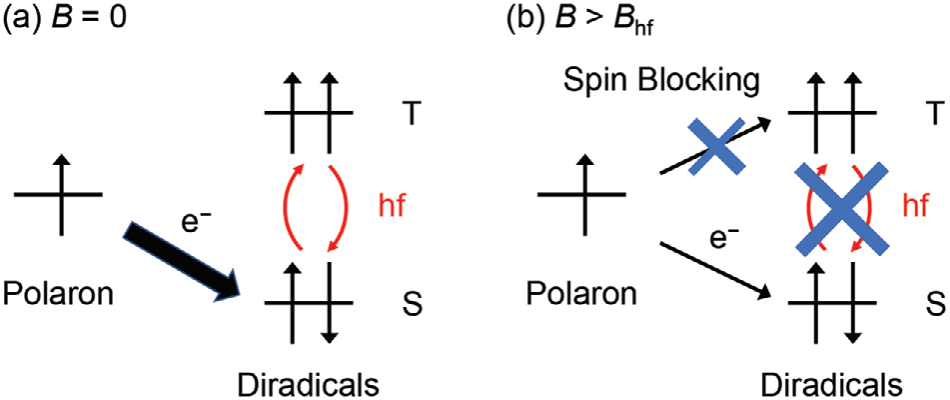
Schematic representation of the possible mechanism of the magnetoconductance with triplet diradicaloids. a) In the absence of the magnetic field, hyperfine field (hf) induces mixing of the spin states of the diradicals to allow the charge transfer from the polaron. b) The magnetic field comparable to or larger than the hf suppresses the mixing of the states, and the spin blocking partially prevents the charge transport to reduce the conductance.

The mechanism of the OMAR effect has been studied extensively. Considering the small magnetic field (<100 mT), Zeeman splitting of the triplet state can be excluded from the possible origin of OMAR. All the proposed mechanisms to date considered that hyperfine fields for spin mixing have an important role under low magnetic fields; rates of the reactions between the two charged spices can be spin‐dependent and the external magnetic field can change these rates through the modulation of the hyperfine coupling, thus the electric currents. Depending on the particle pairs involved in the reaction, the possible mechanisms of OMAR have been categorized into three: polaron pair (bipolaron), electron‐hole pair, and triplet‐polaron pair interactions.^[^ ^50^ ^]^ Although the currently available models do not assume the existence of any diradical species, the similar explanation for OMAR in the diradicaloid is possible based on the following “polaron” model (**Figure** **6**); in the absence of the external magnetic field, the charge transfer from the charge carrier (polaron) to the neutral diradicaloids can be spin‐allowed because the spin mixing through hyperfine fields can lift the spin blocking effect on the triplet diradicals (Figure 6a). When the magnetic fields comparable to or larger than the hyperfine fields are applied, the spin mixing is suppressed resulting in the recovery of the spin blocking for the charge transfer to the triplet states, and thus the decrease in the current (Figure 6b). Since the concentrations of the triplet states in DTBDTCN (2.7% at 120 °C) are much larger than those of the charge carriers, the spin blocking could have large effects on the conduction.

To support this scenario, the MC curves were analyzed by using an empirical fitting function reported by Koopmans group to extract two important parameters: hyperfine field *B*_hf_ and additional broadening *B*_m_.^[^ ^50^, ^51^ ^]^ The MC curves with low magnetic fields of −2 to 2 mT are presented in Figure 5c, which are essential to fit the curves properly to obtain *B*_hf_ accurately. The fitting result shows that *B*_hf_ was constant at around 1.23 mT with a *B*_m_ value of 8‐15 mT at different voltages and temperatures (Figure S23, Supporting Information). The *B*_hf_ value is similar to those reported for materials with the bipolaron model.^[^ ^51^ ^]^ The DFT calculation^[^ ^52^ ^]^ on the model compound gave *B*_hf_ of 1.09 mT for the diradical triplet state of DTBDTCN. These are consistent with the scenario of the polaron model; however, further study is necessary to elucidate the detailed mechanism of OMAR for the diradicaloids.

### 2.8 OFET Characterization

OFET devices containing DTBDTCN were fabricated with a bottom‐gate/top‐contact device configuration. The SiO_2_/Si substrates for device fabrication were modified with cured benzocyclobutene (BCB) films. DTBDTCN thin films were deposited on the substrates by spin‐coating the CHCl_3_ solution, and the films were thermally annealed at 90, 120, 150, and 160 °C. Basically, the thermal annealing enhanced the crystallinity of the films. Detailed analysis of the film structures by atomic force microscopy (AFM), out‐of‐plane X‐ray diffraction (XRD), and grazing incidence wide angle X‐ray scattering (GIWAXS) at each condition are presented and discussed in the Supporting Information.

**Figure 7:**
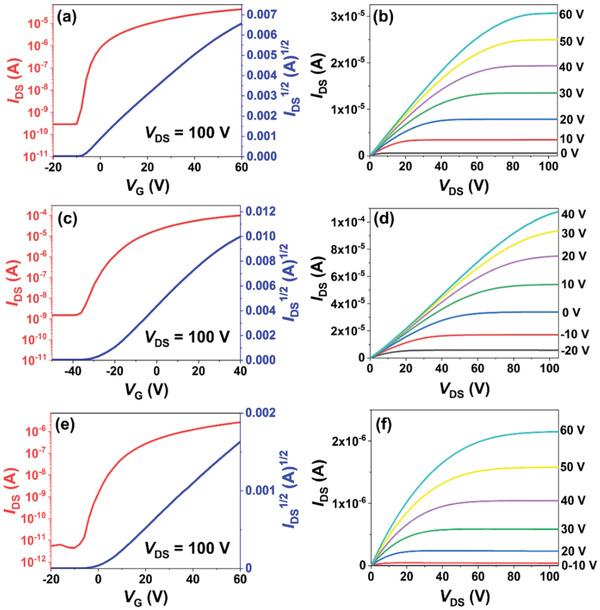
Transfer and output curves of the OFET devices based on a,b) spin‐coated films annealed at 150 °C and c,d) edge‐cast films of DTBDTCN, and e,f) spin‐coated films annealed at 150 °C of DTTTCN.

Unlike other diradical semiconductor materials, which show either ambipolar or p‐type transport properties,^[^ ^34^, ^35^, ^36^, ^37^, ^38^ ^]^ DTBDTCN exhibited n‐type transport property. Moreover, all the devices showed similar performance in air and under an N_2_ atmosphere, indicating the high ambient stability of DTBDTCN devices owing to the deep LUMO energy level.^[^ ^53^ ^]^ **Figure** **7** shows the typical transfer and output curves, and the OFET parameters are summarized in **Table** **1**. Neither the as‐cast film nor the film annealed at 90 °C of DTBDTCN exhibited transport property in the OFETs, whereas the film annealed at 120 °C showed n‐channel characteristics with average electron mobilities of 0.022 cm^2^ V^−1^ s^−1^. The device with the film annealed at 150 °C had a much higher mobility of 0.41 cm^2^ V^−1^ s^−1^ (Figure 7a,b), although increasing the annealing temperature to 160 °C did not change the performance further.

**Table 1:** Effective Electron Mobility (*µ*_e,eff_), Reliability Factor (*r*),^[^ ^57^ ^]^ Current on/off Ratio (*I*_on_/*I*_off_), and Threshold Voltage (*V*_Th_) for OFETs Based on DTBDTCN and DTTTCN

Material	Method	*T* (°C)^a)^	*µ*_e,eff_ (cm^2^ V^−1^ s^−1^)^b)^	*r* ^b)^	*I*_on_/*I*_off_ ^b)^	*V*_Th_ (V)^b)^
DTBDTCN	Spin‐coating	120	0.022 ± 0.004	0.87	10^5^	1.2 to 7.8
		150	0.41 ± 0.02	0.95	10^5^	−5.9 to −12.2
		160	0.39 ± 0.03	0.92	10^5^	−10.1 to −18.3
	Edge‐casting	150	1.01 ± 0.01	0.80	10^4^‐10^5^	−25.6 to −34.5
DTTTCN	Spin‐coating	RT	0.017± 0.002	0.86	10^6^	3.1 to 12.4
		90	0.018± 0.003	0.84	10^6^	2.4 to 11.3
		120	0.030 ± 0.004	0.89	10^6^	1.6 to 8.9
		150	0.033 ± 0.003	0.89	10^6^	0.5 to 6.8
		160	0.031 ± 0.002	0.87	10^6^	0.3 to 6.5

^a)^ Annealing temperature;

^b)^ The average device characteristics were obtained from eight devices, and all devices were measured under ambient conditions.

To optimize the film crystal structure further, DTBDTCN thin films were prepared for the OFET devices by edge‐casting instead of spin‐coating. Edge‐casting is a solution‐crystallizing method that has been used to form single‐crystal films.^[^ ^54^, ^55^, ^56^ ^]^ A droplet of DTBDTCN solution in a mixed solvent of toluene and chlorobenzene (3:1 in v/v) was placed at the edge of a sustaining part on a BCB‐modified SiO_2_/Si substrate (Figure S24a, Supporting Information). Along the evaporation direction of solvent, the domains grew on top of substrate in the direction of OFET channel (Figure S24b, Supporting Information), which allowed efficient charge transport. Although edge‐casting did not form single‐crystal DTBDTCN thin films, the films did show outstanding n‐channel OFET performance with an average electron mobility of 1.01 cm^2^ V^−1^ s^−1^, which was higher than those of the spin‐coated films (Figure 7c,d, Table 1) and is the highest reported electron mobility for OFETs based on diradicaloids to date.

OFET devices based on spin‐coated DTTTCN thin films were fabricated with the same method for DTBDTCN. Although DTTTCN devices also showed air‐stable electron transport property, their optimal performance was much lower compared to that of DTBDTCN devices (Figure 7e,f, Table 1). The as‐cast film of DTTTCN exhibited n‐channel characteristics with average electron mobilities of 0.017 cm^2^ V^−1^ s^−1^. Unlike DTBDTCN, the effect of thermal annealing on DTTTCN device performance was small. The electron mobility of spin‐coated DTTTCN thin film was slightly enhanced after thermal annealing at 90, 120, 150, and 160 °C, and a highest mobility of 0.033 cm^2^ V^−1^ s^−1^ was observed when the thin films were thermal annealed at 150 °C. In addition, edge‐casting was not applicable to the deposition of DTTTCN film, because the domains of DTTTCN were unable to grow in the direction of OFET channel on top of substrate with this method.

### 2.9 Comparison with other organic semiconductors that show OMAR

OMAR effects reported for the common organic semiconductors are summarized in **Table** **2** and compared with those of DTBDTCN in this study. None of the previously reported materials has diradical properties. The particularly large positive MC has been observed for Alq_3_ and PFO at room temperature (25% and 16%, respectively).^[^ ^13^, ^14^ ^]^ A notable exception is m‐MTDATA:3TPYMB mixture system that showed extremely high MC of over +1000% after device conditioning by constant current application, in which the involvement of triplet excited states formed by charge transfer at the materials interface was proposed.^[^ ^15^ ^]^ In comparison with these strong positive MC effects, the negative MC effects have been observed with the lower values and often along with the positive MC effects depending on the measurement conditions. The MC of −5.3% observed for DTBDTCN at room temperature is the largest negative MC among these organic semiconductors. The temperature effects on OMAR for the common materials are hard to find in the literatures and limited only to the low temperatures. In contrast, DTBDTCN showed the strong temperature dependence of MC that was ascribed to the triplet concentrations and the record high negative MC of −19.4% was observed at 120 °C. This seems a unique feature of the OMAR effects based on the thermally activated triplet diradicals. The negative MC can be explained by the spin‐blocking mechanism between the polarons and the triplet states as described above. The operation voltage for DTBDTCN (3.2 V) is relatively low compared to those for the other materials with the significant positive MC (e.g., Alq_3_ at 13 V). This could be attributed to the high electron mobility of DTBDTCN which increases the charge carrier density at low voltages in the diode devices. However, the relationship between the electronic properties and the OMAR effects are yet to be elucidated by systematic investigations on various semiconducting diradicaloids.

**Table 2:** Comparison of OMAR effects in common organic semiconductor and **DTBDTCN**

Materials	MC_max_ [%]^a)^	*V* [V]^b)^	*B*_max_ [mT]^c)^	*T* [K]^d)^	Reference
Alq_3_	+25	13	100	298	^[^ ^13^ ^]^
PFO	+16	5	100	300	^[^ ^14^ ^]^
m‐MTDATA :3TPYMB	> +1000	30	300	294	^[^ ^15^ ^]^
RR‐P3HT	+1.8	5.4	100	298	^[^ ^16^ ^]^
	−3.9	14	300		
MDMO‐PPV	+2.2	2.3	60	298	^[^ ^17^ ^]^
	‐0.5	1.6			
PCBM	−3	3.1	60	298	^[^ ^18^ ^]^
PVK	1.2 and −1.5	–^e^	300	298	^[^ ^20^ ^]^
Pentacene	+2	3.3	90	100	^[^ ^21^ ^]^
	−2	1.2		300	
**DTBDTCN**	−5.3	3.2	200	298	This work
	−19.4			393	

^a)^ Maximum positive and negative MC. All reported magnetoresistance (MR) values were converted to MC by the equation of MC = −MR/(MR+1);

^b)^ Applied constant voltages to monitor the current;

^c)^ Maximum applied magnetic fields to observe MC_max_;

^d)^ Temperatures for MC measurements;

^e)^ Constant current condition.

## 3 Conclusion

The diradicaloid quinoidal semiconductor DTBDTCN shows large OMAR effects in diode devices and the high electron transport property in OFETs. Moreover, MC is directly related to the concentration of triplet diradicals, and thus it can be a unique property of diradicaloid semiconductors. In contrast, DTTTCN with the diradical character but no accessible triplet state showed no OMAR effect and much lower OFET performance compared to DTBDTCN. The unprecedented link between the diradical character and OMAR effect paves the way to organic spintronics based on triplet diradical materials. This finding may also spark the development of new diradicaloid materials with high stability and different spin‐state energetics for the application in magnetoresistive sensors.

## 4 Experimental Section

### Materials and Characterizations

BCB was purchased from Dow Chemical Company. The other commercial reagents were purchased from FUJIFILM Wako Pure Chemical (Japan), TCI Chemicals Co. (Japan), or Sigma‐Aldrich (USA), and used without further purification. The synthetic details and characterization of DTBDTCN and DTTTCN are described in the Supporting Information. All air‐or moisture‐sensitive reactions were performed under N_2_ by Schlenk techniques. ^1^H and ^13^C NMR spectra were recorded on a 300 MHz spectrometer (JNM‐AL300, JEOL). VT ^1^H NMR spectra were recorded on a 400 MHz spectrometer (JNM‐ECZ400, JEOL). NMR data are presented as follows: chemical shift in ppm (*δ*), multiplicity (s = singlet, d = doublet, t = triplet, and m = multiplet), coupling constant in Hz, and integration. HRMS was carried out on a mass spectrometer (JMS‐T100GCV, JEOL). TGA was performed on a thermal gravimetric analyzer (TG 8120, Rigaku) under N_2_ at a heating rate of 10°C min^−1^, heating from room temperature to 500 °C. DSC was conducted on a differential scanning calorimeter (DSC 8230, Rigaku) under an N_2_ flow at a heating rate of 10 °C min^−1^ in a temperature range from room temperature to 250 or 300 °C. The UV‐vis‐NIR absorption spectra were recorded on a spectrophotometer (V‐670, JASCO). CV measurement was carried out on an electrochemical instrument (HSV‐100, HOKUTO DENKO) in anhydrous dichloromethane with 0.1 m tetrabutylammonium hexafluorophosphate as the supporting electrolyte under an N_2_ atmosphere. The three‐electrode cell for CV was equipped with a platinum disk working electrode, a platinum wire counter electrode, and an Ag/AgNO_3_ reference electrode. The scan rate was 0.05 V s^−1^, and the potential was calibrated against the ferrocene/ferrocenium couple. The film thickness was measured with a surface profilometer (Dektak 6 m, ULVAC‐PHI). AFM images were recorded on a scanning probe microscope (5400, Agilent Technologies) in tapping mode. XRD measurements were carried out on an X‐ray diffractometer (Smartlab, Rigaku) with Cu K*α* radiation (*λ* = 0.154 nm). The GIWAXS analysis was performed at beamline BL46XU of SPring‐8, Japan. The X‐ray energy for GIWAXS was 12.398 keV (wavelength: 0.10002 nm) and the incident angle of the measurements was fixed at 0.12° using a Huber diffractometer.

### ESR Sample Preparation and Measurement

A quartz ESR tube containing DTBDTCN powder (2.0 mg, 1.923 × 10^−6^ mol) was evacuated for 4 h, and then flame sealed. VT‐ESR measurements of DTBDTCN were performed on an ESR spectrometer (JES‐X320, JEOL) equipped with a variable temperature controller (ES‐13060DVT5, JEOL). A TEMPOL solution (3.309 × 10^−6^ m) in anhydrous toluene with the spin number of 1.094 × 10^15^ was used as a standard sample. The integration of the ESR signals was calibrated with Mn marker peaks. The double integral of the DTBDTCN signal at variable temperatures relative to the TEMPOL signal was used to quantify the spin concentrations in DTBDTCN. Fitting of the VT ESR data was performed with the Bleaney‐Bowers equation reported in the literature.^[^ ^58^ ^]^ The ESR sample of DTTTCN was prepared and measured with the same methods as those for DTBDTCN.

### OMAR Device Fabrication and Measurement

After cleaning and UV‐O_3_ treatment, ITO substrates were spin‐coated with PEDOT:PSS at 4000 rpm for 30 s. The active layer was deposited by spin‐coating a 5 mg mL^−1^ chloroform solution of DTBDTCN at 1000 rpm for 30 s in a glovebox, followed by thermal annealing at 150 °C for 30 min. Films with thicknesses of 13–91 nm were obtained by changing the solution concentration from 1.5 to 8 mg mL^−1^. Subsequently, the thin films were transferred into a vacuum evaporator connected to the glovebox, and the cathode layers of Ca (20 nm) and Al (80 nm) were deposited sequentially. The device was returned to the glovebox and sealed with a glass cap and a photocurable resin. For the electron‐only devices, the ITO surface was spin‐coated with a PEIE buffer layer ≈10 nm thick. DTBDTCN film was prepared in the same way for the bipolar devices. Then, Ag (60 nm) or Ca (20 nm)/Al (80 nm) was deposited on top of DTBDTCN film by vacuum evaporation. For the hole‐only device, PEDOT:PSS layer and DTBDTCN film were prepared in the same way for the bipolar device, but MoO_3_ (10 nm)/Ag (60 nm) was used as the cathode electrode instead of Ca/Al. The device measurements were performed on a variable‐temperature heating stage placed between the poles of an electromagnet. The devices were driven at a constant voltage using a source meter (2611A, Keithley), and the current was measured while sweeping the magnetic field, *B*. Variable temperature measurements were performed in a temperature range of 25–120 °C. Obvious deterioration of the devices was observed at above 120 °C. The fabrication and measurement of the DTTTCN devices were the same as those for the DTBDTCN bipolar devices.

### OFET Device Fabrication and Measurement

OFETs were fabricated in the bottom‐gate/top‐contact configuration. To eliminate charge traps in SiO_2_, the cleaned SiO_2_/Si substrate was covered with an insulating layer of BCB by spin‐coating of a 5 vol% BCB solution in mesitylene followed by thermal annealing at 250 °C for 3 h in a glovebox. The total capacitance of the SiO_2_ and BCB layer was 9.7 nF cm^−2^. The semiconducting layer was deposited on the BCB‐treated substrate by spin‐coating a 5 mg mL^−1^ chloroform solution of DTBDTCN or DTTTCN at a speed of 1000 rpm for 30 s, followed by thermal annealing at different temperatures for 30 min. All preparation procedures were carried out in a glovebox. For the edge‐cast film, the edge‐casting method shown in Figure S16a, Supporting Information was performed on a horizontal BCB‐modified substrate in air. A solution of DTBDTCN (0.5 mg mL^−1^, 100 µL) in a mixed solvent of toluene and chlorobenzene (3:1 in v/v) was dropped on the edge of a holding piece. The edge‐cast film was obtained after solvent evaporation, and then the film was thermally annealed at 150 °C for 30 min in a glovebox. After the formation of the semiconductor films, top‐contact electrodes of Au (30 nm) and Ag (40 nm) were deposited sequentially by vacuum evaporation through a metal shadow mask. The channel length and width were 200 and 1000 µm, respectively. OFET devices were measured under ambient conditions. Different source meters were used to measure the source−drain current (6430, Keithley) and gate leakage current (2400, Keithley). The effective charge carrier mobility was estimated in the saturation region according to the literature^[^ ^57^ ^]^ considering non‐ideality in the transfer characteristics.

### Theoretical Calculations

Quantum chemical calculations were performed with Gaussian 16 software package based on DFT. First, the structures of the model compound were optimized at the B3LYP/6‐31G(d,p) level of theory with the restricted and unrestricted spin configurations for the closed‐shell and the open‐shell structures, respectively, with the designated spin states. The diradical character value (*y*_0_) of the open‐shell singlet structure was calculated for the optimized structure with UCAM‐B3LYP/6‐31G(d,p) by using the occupation numbers in the HOMO and LUMO according to the literature.^[^ ^45^ ^]^ The hyperfine field of the model compound for the open‐shell triplet structure was calculated from the isotropic Fermi contact coupling constants in the Gaussian output file according to the literature.^[^ ^52^ ^]^

## Conflict of Interest

The authors declare no conflict of interest.

## Supporting information

Supporting Information

Supplemental Video 1

## Data Availability

The data that support the findings of this study are available in the supplementary material of this article.
