# Supplementary material for: Essential Role of Triplet Diradical Character for Large Magnetoresistance in Quinoidal Organic Semiconductor with High Electron Mobility
Source: Adv Sci (Weinh). 2022 Mar 28;9(16):2201045. doi: 10.1002/advs.202201045 (PMC9165494; doi:10.1002/advs.202201045)
Supplement: Supplementary file 1 — Supporting Information [file ADVS-9-2201045-s001.pdf]

ADVANCED SCIENCE
Open Access

Supporting Information

for *Adv. Sci.*, DOI 10.1002/advs.202201045

Essential Role of Triplet Diradical Character for Large Magnetoresistance in Quinoidal Organic Semiconductor with High Electron Mobility

*Chao Wang, Hua Hao and Keisuke Tajima**

Supporting Information

Essential Role of Triplet Diradical Character for Large Magnetoresistance in Quinoidal Organic Semiconductor with High Electron Mobility

*Chao Wang, Hua Hao, Keisuke Tajima**
RIKEN Center for Emergent Matter Science (CEMS), 2-1 Hirosawa, Wako, Saitama 351-0198, Japan

Synthesis of DTBDTCN

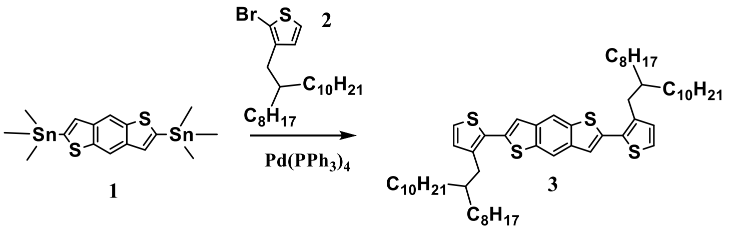

*Synthesis of 2,6-bis(3-(2-octyldodecyl)thiophen-2-yl)benzo[1,2-b:4,5-b']dithiophene (**3**).* **1** (0.56 g, 1.09 mmol) and **2** (1.21 g, 2.73 mmol) were dissolved in dry DMF (10 mL) under N_2. $Pd(PPh_3)_4$ (0.13 g, 0.109 mmol) was added to the solution and the resulting mixture was stirred at 120 °C for 10 h under N_2. After cooling to room temperature, the solvent was removed under reduced pressure, and the residue was purified by column chromatography on silica gel (*n*-hexane) to give **3** as a pale-yellow oil (0.78 g, yield 78%). ^{1}H NMR (CD_2Cl_2, 300 MHz): δ 8.18 (s, 2H), 7.37 (s, 2H), 7.30 (d, J =5.2 Hz, 2H), 6.98 (d, J = 5.2 Hz, 2H), 2.84 (d, J = 7.2 Hz, 4H), 1.72 (m, 2H), 1.19–1.26 (m, 64 H), 0.83–0.88 (m, 12H). ^{13}C NMR (CD_2Cl_2, 300 MHz): δ 141.37, 138.73, 138.42, 138.06, 131.9, 125.70, 122.37, 116.90, 39.81, 34.61, 34.20, 32.71, 30.79, 30.43, 30.39, 30.10, 27.21, 23.46, 14.64. HRMS (FD) m/z calcd. for $C_{58}H_{90}S_4$: 914.59253; found: 914.59287.

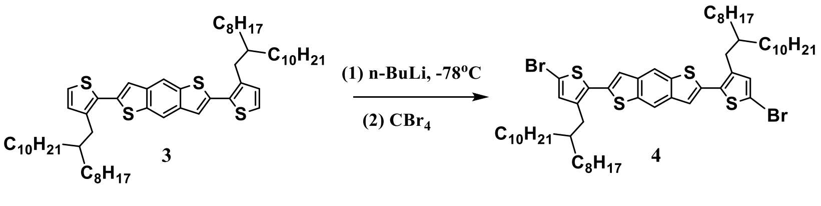

*Synthesis of 2,6-bis(5-bromo-3-(2-octyldodecyl)thiophen-2-yl)benzo[1,2-b:4,5-b']dithiophene (**4**).*

3 (302 mg, 0.33 mmol) was dissolved in anhydrous THF (10 mL), and then a solution of *n*-butyllithium in hexane (1.6 M, 0.53 mL) was added dropwise to the solution at −78 °C. The reaction was stirred at −78 °C for 1 h, and then a solution of carbon tetrabromide (328 mg, 0.99 mmol) in anhydrous THF (6 mL) was added. The mixture was stirred overnight at room temperature, and then hydrolyzed with water and extracted with $CHCl_3$. The combined organic phases were washed with brine and dried over anhydrous Na_2SO_4. After evaporation of the solvent, the residue was purified by column chromatography on silica gel with *n*-hexane/CH_2Cl_2 (9:1) to give **4** as a pale-yellow solid (257 mg, yield 72%). ^{1}H NMR ($CDCl_3$, 300 MHz): δ 8.15 (s, 2H), 7.28 (s, 2H), 6.90 (s, 2H), 2.74 (d, J = 7.1 Hz, 4H), 1.66 (m, 2H), 1.18–1.22 (m, 64H), 0.83–0.89 (m, 12H). ^{13}C NMR ($CDCl_3$, 300 MHz): δ 141.39, 138.06, 136.30, 133.76, 133.05, 122.36, 116.61, 112.10, 39.30, 34.14, 33.63, 32.21, 30.26, 29.93, 29.89, 29.64, 26.71, 22.97, 14.39. HRMS (FD) *m/z* calcd. for $C_{58}H_{88}Br_2S_4$: 1072.41151; found: 1072.40797.

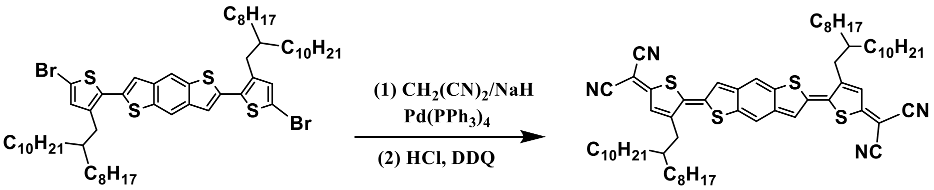

4 **DTBDTCN**

Synthesis of ***DTBDTCN***. Sodium hydride (60% in oil, 56 mg, 1.4 mmol) was added to a degassed solution of malononitrile (46 mg, 0.7 mmol) in anhydrous dimethoxyethane (10 mL) at 0 °C under an N_2 atmosphere, and the resulting suspension was stirred at room temperature for 30 min to give a solution of the malononitrile anion. **4** (0.25 g, 0.233 mmol) and $Pd(PPh_3)_4$ (80.9 mg, 0.07 mmol) were dissolved in dimethoxyethane (10 mL), and then the mixture was heated under reflux for 40 min, followed by the slow addition of the malononitrile anion solution with a syringe. The mixture was refluxed for 7 h. After cooling to room temperature, hydrochloric acid (2 M, 20 mL) was added with cooling in an ice bath. The resulting mixture was extracted with CH_2Cl_2, and DDQ (78.6 mg, 0.35 mmol) was added to the solution. After stirring at room temperature for 2 h, the reaction mixture was concentrated in vacuo, and purified by column chromatography on silica gel with toluene/*n*-hexane followed by recrystallization in $CHCl_3$/MeOH to give **DTBDTCN** as a brown solid (78 mg, 32% yield). ^{1}H NMR (CD_2Cl_2, 400 MHz, −60 °C): δ 7.59 (s, 2H), 7.24 (s, 2H), 7.10 (s, 2H), 2.78 (d, J = 7.2

Hz, 4H), 1.86 (m, 2H), 1.26–1.37 (m, 64H), 0.86 ‑ 0.89 (m, 12H). HRMS (FD) *m/z* calcd. for $C_{64}H_{88}N_4S_4$: 1039.58135 [M^+]; found: 1039.58096.

Notably, an isomeric byproduct with the alkyl chains on the same side of quinoid core was also obtained after the reaction. The ratio of **DTBDTCN** to this byproduct was about 5:1. **DTBDTCN** was separated from the byproduct by column chromatography because of the polarity difference. There was no interconversion observed between the isomers at room temperature.

Synthesis of DTTTCN

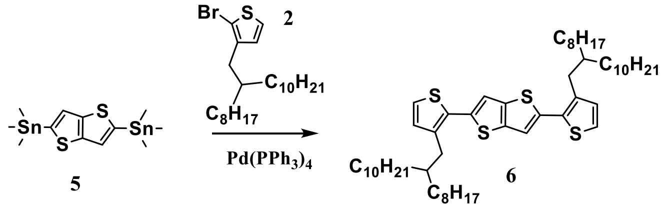

*Synthesis of 2,5-bis(3-(2-octyldodecyl)thiophen-2-yl)thieno[3,2-b]thiophene (**6**).* **5** (0.22 g, 0.47 mmol) and **2** (0.55 g, 1.23 mmol) were dissolved in dry DMF (10 mL) under N_2. $Pd(PPh_3)_4$ (54 mg, 0.047 mmol) was added to the solution and the resulting mixture was stirred at 120 °C for 10 h under N_2. After cooling to room temperature, the solvent was removed under reduced pressure, and the residue was purified by column chromatography on silica gel (*n*-hexane) to give **6** as a pale-yellow oil (0.29 g, yield 71%). ^{1}H NMR (CD_2Cl_2, 300 MHz): 7.25 (s, 2H), 7.23 (d, *J* =5.2 Hz, 2H), 6.96 (d, *J* = 5.2 Hz, 2H), 2.75 (d, *J* = 7.2 Hz, 4H), 1.70 (m, 2H), 1.23 (m, 64 H), 0.84–0.88 (m, 12H). ^{13}C NMR (CD_2Cl_2, 300 MHz): δ 140.73, 140.10, 138.63, 132.02, 131.56, 125.17, 119.26, 39.80, 34.49, 34.23, 32.72, 30.79, 30.44, 30.39, 30.16, 27.23, 23.47, 14.66. HRMS (FD) *m/z* calcd. for $C_{54}H_{88}S_4$: 864.57688; found: 864.57656.

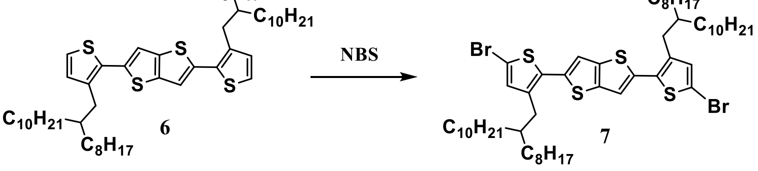

*Synthesis of 2,5-bis(5-bromo-3-(2-octyldodecyl)thiophen-2-yl)thieno[3,2-b]thiophene (**7**).* **6** (260 mg, 0.3 mmol) and NBS (111 g, 0.62 mmol) were added to a mixture of $CHCl_3$ (10 mL) and acetic

acid (5 mL) at 0 °C. The mixture was allowed to warm to room temperature and stirred overnight. The reaction mixture was quenched with water and extracted with $CHCl_3$. The organic layer was dried over anhydrous $MgSO_4$. After evaporation of the solvent, the residue was purified by column chromatography on silica gel (hexane) to give **7** as a pale-yellow oil (236 mg, yield 77%). ^{1}H NMR (CD_2Cl_2, 300 MHz): 7.21 (s, 2H), 6.93 (s, 2H), 2.69 (d, J = 7.2 Hz, 4H), 1.65 (m, 2H), 1.22-1.24 (m, 64 H), 0.85–0.89 (m, 12H). ^{13}C NMR (CD_2Cl_2, 300 MHz): δ 141.64, 140.32, 137.48, 134.19, 133.51, 119.75, 111.84, 39.76, 34.43, 34.12, 32.74, 30.73, 30.46, 30.37, 30.16, 27.18, 23.48, 14.66. HRMS (FD) *m/z* calcd. for $C_{54}H_{86}Br_2S_4$: 1020.39791; found: 1020.39807.

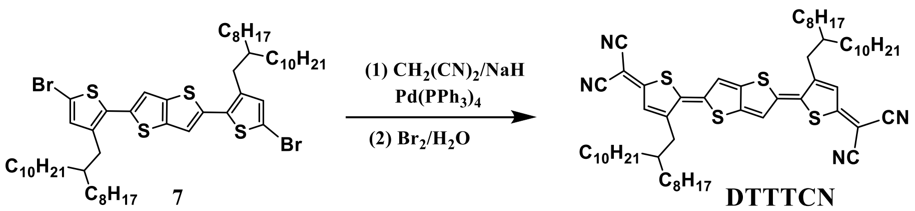

*Synthesis of **DTTTCN***. Sodium hydride (60% in oil, 35 mg, 0.88 mmol) was added to a degassed solution of malononitrile (29 mg, 0.44 mmol) in anhydrous dimethoxyethane (10 mL) at 0 °C under an N_2 atmosphere, and the resulting suspension was stirred at room temperature for 30 min to give a solution of the malononitrile anion. **7** (151 g, 0.148 mmol) and $Pd(PPh_3)_4$ (50.8 mg, 0.044 mmol) were dissolved in dimethoxyethane (10 mL), and then the mixture was heated under reflux for 40 min, followed by the slow addition of the malononitrile anion solution with a syringe. The mixture was refluxed for 5 h. After cooling to room temperature, saturated bromine water was added and stirred for 1h. The resulting mixture was extracted with $CHCl_3$ and concentrated in vacuo. The crude product was purified by column chromatography on silica gel with CH_2Cl_2/*n*-hexane followed by recrystallization in $CHCl_3$/MeOH to give **DTBDTCN** as a brown solid (64 mg, 44% yield). ^{1}H NMR ($CDCl_3$, 300 MHz): δ 7.20 (s, 2H), 7.08 (s, 2H), 2.76 (d, J = 6.9 Hz, 4H), 1.83 (m, 2H), 1.28–1.35 (m, 64H), 0.86–0.90 (m, 12H). ^{13}C NMR ($CDCl_3$, 300 MHz): δ 167.43, 155.92, 152.50, 145.63, 136.54, 133.16, 121.63, 114.55, 113.70, 37.53, 36.38, 33.35, 31.97, 29.98, 29.69, 29.63, 29.41, 26.29, 22.74, 14.16. HRMS (FD) *m/z* calcd. for $C_{60}H_{86}N_4S_4$: 990.57353 [M^+]; found: 990.57377.

Thin-film Morphology

Atomic force microscopy (AFM) was used to investigate the surface morphologies of the films. The **DTBDTCN** spin-coated thin films had small crystallite grains with the size of 100–300 nm (**Figure S25**). After thermal annealing at 120 °C, flat domains around 500 nm in size appeared, and the domains grew gradually as the annealing temperature was increased to 160 °C. A step height of about 3 nm was observed between the domains after annealing at 150 °C (**Figures S26**), which was similar to the size of the molecule. The edge-cast film had much larger crystal domains than the spin-coated films with sizes larger than 10 µm (**Figure S25f**). In contrast, the **DTTTCN** spin-coated films had much smaller domains (**Figure S27**). The domain size of all **DTTTCN** films is below 300 nm, and only slight change in domain size of **DTTTCN** films before and after annealing could be observed.

Figure S28 shows the out-of-plane X-ray diffraction (XRD) patterns of thin films of **DTBDTCN** and **DTTTCN**. For the **DTBDTCN** spin-coated thin films, both the as-cast and the film annealed at 90 °C showed low crystallinity with only one weak diffraction peak (**Figure S28a**). The diffraction intensity and the number of the higher-order diffraction peaks increased substantially after annealing above 120 °C. Among the spin-coated films of **DTBDTCN**, the film annealed at 150 °C showed the highest order with eight diffraction peaks and strong diffraction intensity, which was consistent with its high OFET performance. The lamellar *d*-spacing estimated from the 001 diffraction peak (2θ = 2.92°) was 30.2 Å, which roughly agreed with the molecule size of **DTBDTCN** in the long-branched alkyl direction and with the step height in the AFM images. Consistent with the highest electron mobility in the device, the edge-cast film of **DTBDTCN** showed the same crystal phase but higher diffraction intensities compared with the spin-coated film annealed at 150 °C. For the spin-coated **DTTTCN** films, the effect of thermal annealing on film order was not significant, although the diffraction intensity was gradually enhanced with increasing annealing temperature from 90 to 150 °C (**Figure S28b**). The **DTTTCN** film annealed at 150°C showed the highest order with only four diffraction peaks, and the diffraction intensity was much lower relative to that of **DTBDTCN** film. This was consistent with the much poorer OFET performance of **DTTTCN** compared to **DTBDTCN**. The lamellar *d*-spacing of **DTTTCN**

calculated from the 001 diffraction peak (2θ = 3.02°) was 29.2 Å, which was similar to the molecule size of **DTTTCN** in the long-branched alkyl direction.

Grazing-incidence wide-angle X-ray scattering (GIWAXS) patterns of **DTBDTCN** and **DTTTCN** were also investigated. For **DTBDTCN** films, both the as-cast film and the film annealed at 90 °C exhibited a typical disordered face-on orientation, whereas the film annealed at 120 °C showed a mixture of the face-on and edge-on orientations (**Figure S29**). After annealing at 150 and 160 °C, the **DTBDTCN** films showed higher-order peaks that matched the edge-on orientation of the molecules (**Figure S29d** and **e**). Generally, the edge-on orientation allows efficient charge transport in OFET because the π-planes of the molecules stack along the charge transport direction in the channel.[1] The line profile along the q_{xy} axis (in-plane) of the GIWAXS pattern for the film annealed at 150 °C (**Figure S30**) revealed a small π-stacking diffraction with a distance of 3.34 Å, suggesting the close packing of **DTBDTCN** molecules. This small π-stacking distance is beneficial for charge transport and may be related to the intermolecular spin-spin interaction of **DTBDTCN** diradicals, as suggested in the literature.[2] The edge-on orientation and the close intermolecular packing could explain the high OFET performance of the **DTBDTCN** film annealed at 150 and 160 °C.

The GIWAXS of both as-cast and annealed films of **DTTTCN** showed mainly edge-on orientation, and the arc reflection shape indicated that **DTTTCN** film also had a small population of face-on domains (**Figure S31**). Thermal annealing did not lead to great change in GIWAXS pattern of **DTTTCN**, which agreed well with the small annealing effect on thin film absorption spectra, XRD patterns and OFET performance. By taking the line profiles along the q_{xy} axes of the GIWAXS image (**Figure S32**), the π-stacking distance of **DTTTCN** was calculated to be 3.66 Å, larger than that of **DTBDTCN**, which was also consistent with the lower OFET performance of **DTTTCN**. These results indicate that **DTBDTCN** can show stronger molecular packing and higher charge transport property compared to **DTTTCN**.

Supplementary Tables and Figures

Table S1. The energy differences between the closed-shell singlet, open-shell singlet, and open-shell triplet states, and diradical character values (y_0) for **DTBDTCN**, **DTTTCN** and the diradicaloid molecules in the literatures.

Materials	Calcd. ΔE(CS−OS)[a]	Calcd. $\Delta E(T_1-S_0)$[b]	$\Delta E(T_1-S_0)$[c]	y_0	Reference
**DTBDTCN**	0.147 eV (3.39 kcal/mol)	0.114 eV (2.63 kcal/mol)	0.14 eV (3.23 kcal/mol)	0.594	This work
**DTTTCN**	0.0065 eV (0.149 kcal/mol)	0.278 eV (6.41 kcal/mol)	-	0.186	This work
Heptazethrene derivative	5.8 kcal/mol	7.5 kcal/mol	-	-	[3]
Tri-p-QM1	5.21 kcal/mol	1.83 kcal/mol	2.12 kcal/mol	-	[4]
4QT-TPD	-	3.1 kcal/mol	2.76 kcal/mol	0.61	[5]
QTICN	4.02 kcal/mol	3.90 kcal/mol	-	0.67	[6]

[a]The calculated energy difference between the closed-shell singlet and open-shell singlet states. [b]The calculated energy difference between the open-shell triplet and open-shell singlet states. [c]The experimetally determined energy difference between the open-shell triplet and open-shell singlet states.

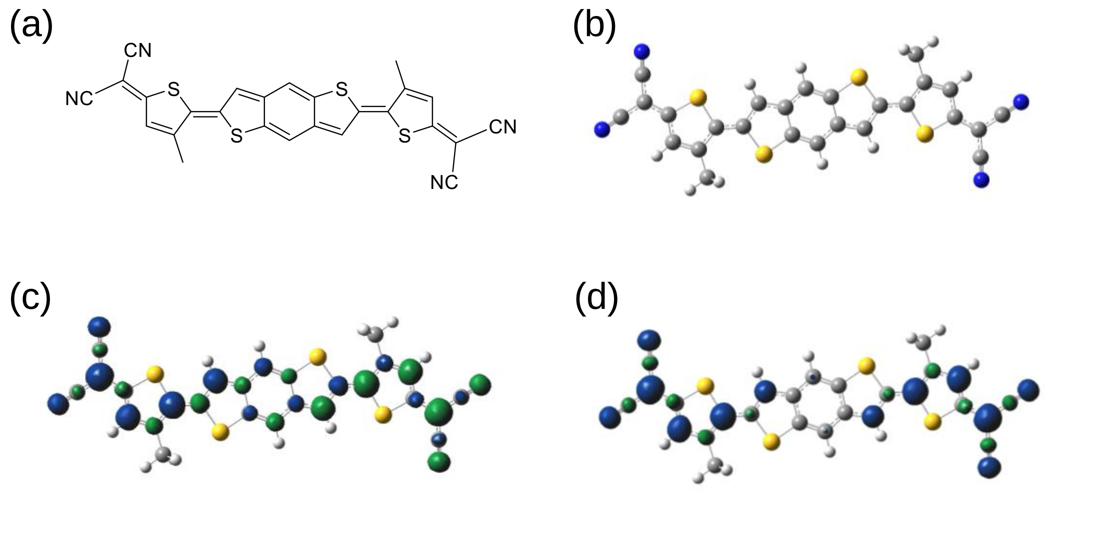

Figure S1. (a) Chemical structure of the model compound for **DTBDTCN** and the optimized structures with the spin density distributions for (b) closed-shell, (c) open-shell singlet, and (d) open-shell triplet states. The calculations are performed at B3LYP/6-31G(d,p) level of theory. The isovalue of the surfaces for the spin density is 0.004.

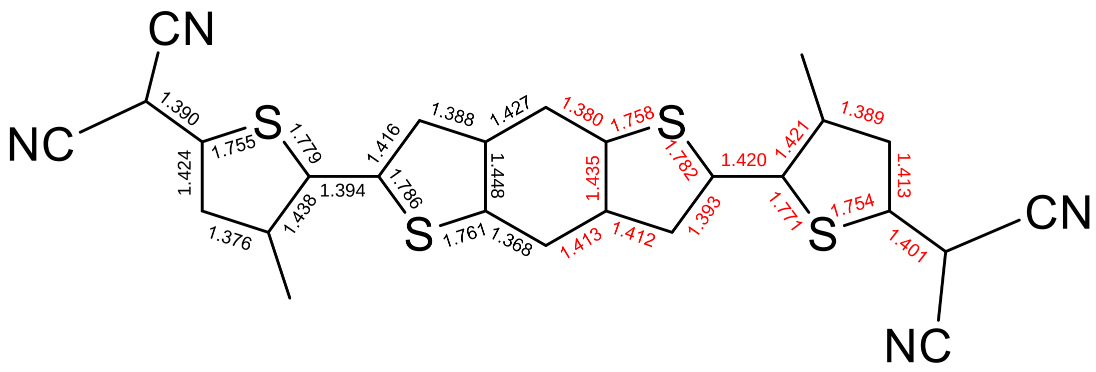

Figure S2. Comparison of the bond lengths for the optimized structures of the closed-shell (black) and open-shell (red) singlets in the **DTBDTCN** model compound.

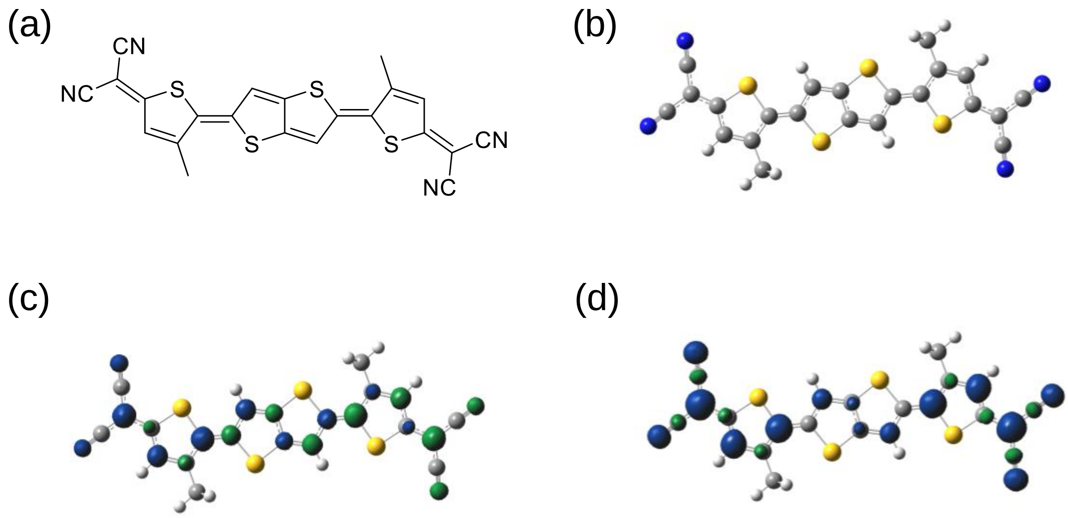

Figure S3. (a) Chemical structure of the model compound for **DTTTCN** and the optimized structures with the spin density distributions for (b) closed-shell, (c) open-shell singlet, and (d) open-shell triplet states. The calculations are performed at B3LYP/6-31G(d,p) level of theory. The isovalue of the surfaces for the spin density is 0.004.

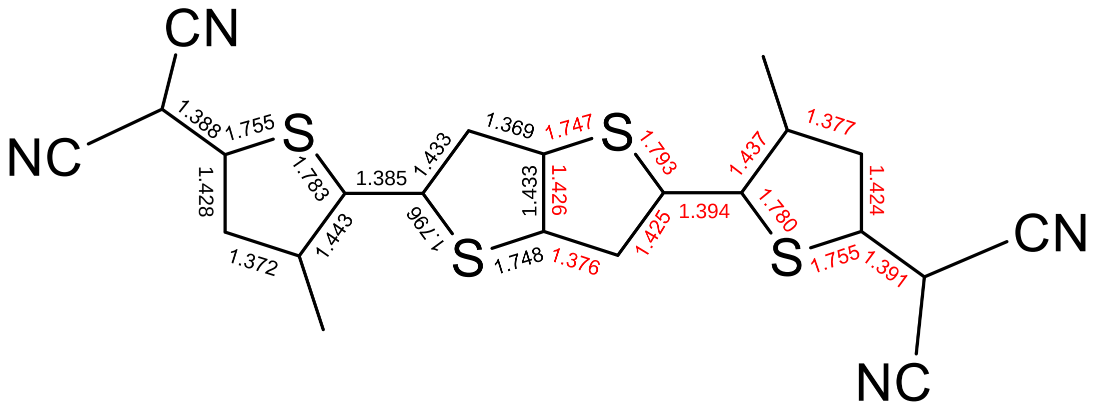

Figure S4. Comparison of the bond lengths for the optimized structures of the closed-shell (black) and open-shell (red) singlets in the **DTTTCN** model compound.

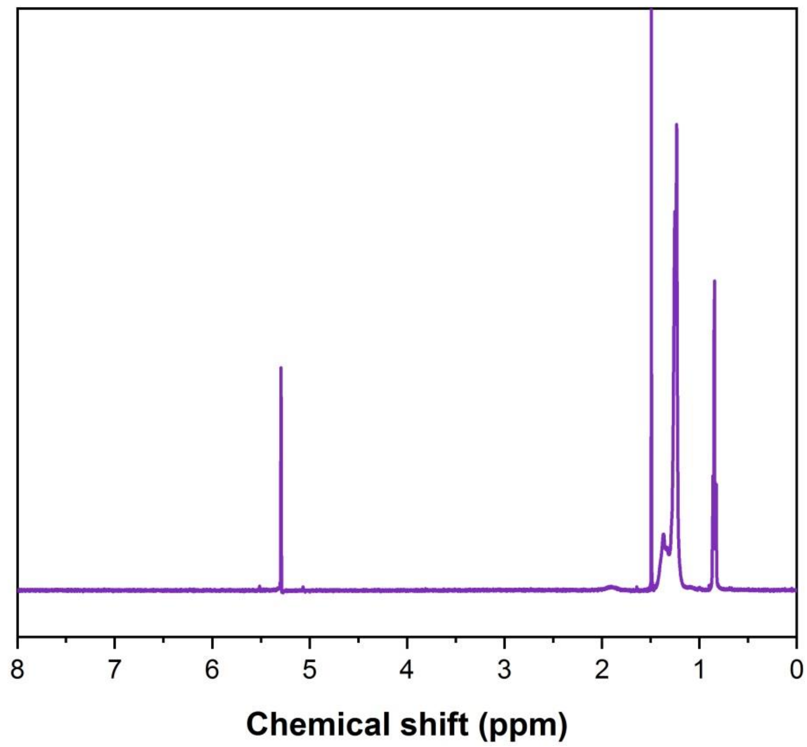

Figure S5. ^{1}H NMR spectrum of **DTBDTCN** in CD_2Cl_2 at 25 °C (full range).

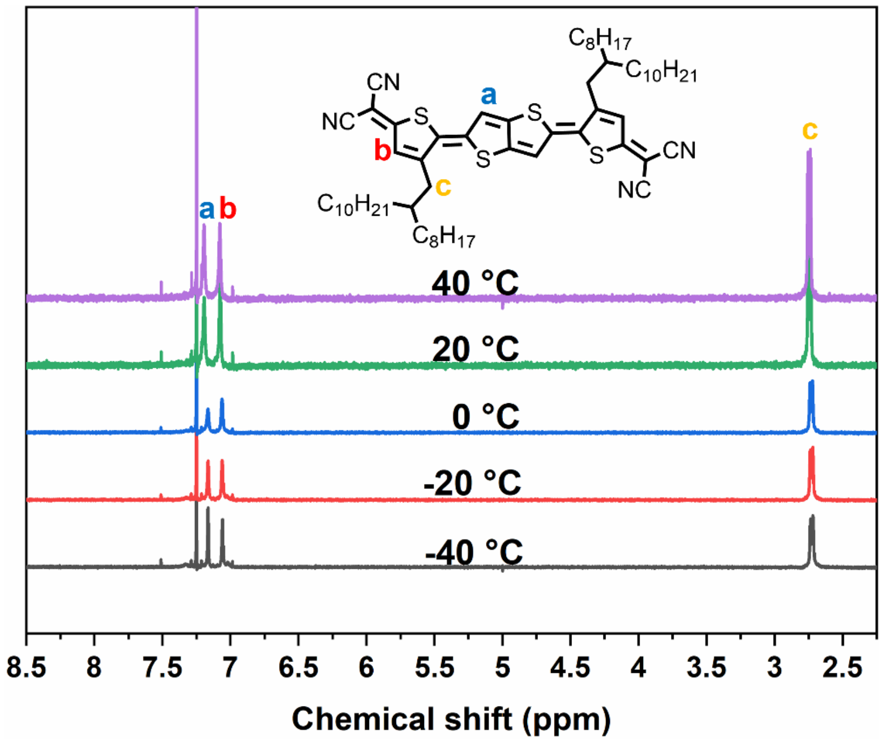

Figure S6. VT ^{1}H NMR spectra of **DTTTCN** in $CDCl_3$ and assignment of the protons in the quinoidal π-conjugated backbone and α-methylene of the alkyl side chains.

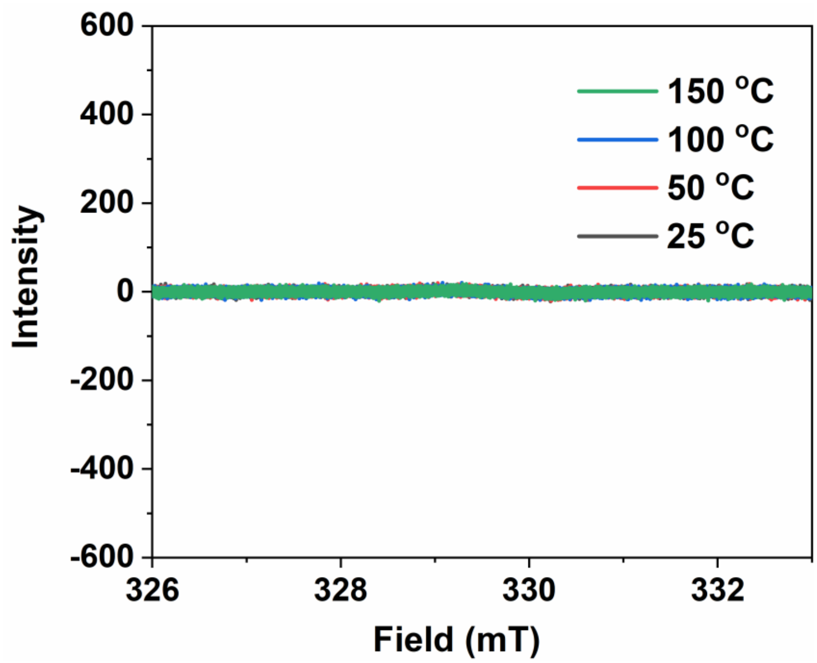

Figure S7. VT-ESR spectra of **DTTTCN** in the solid state.

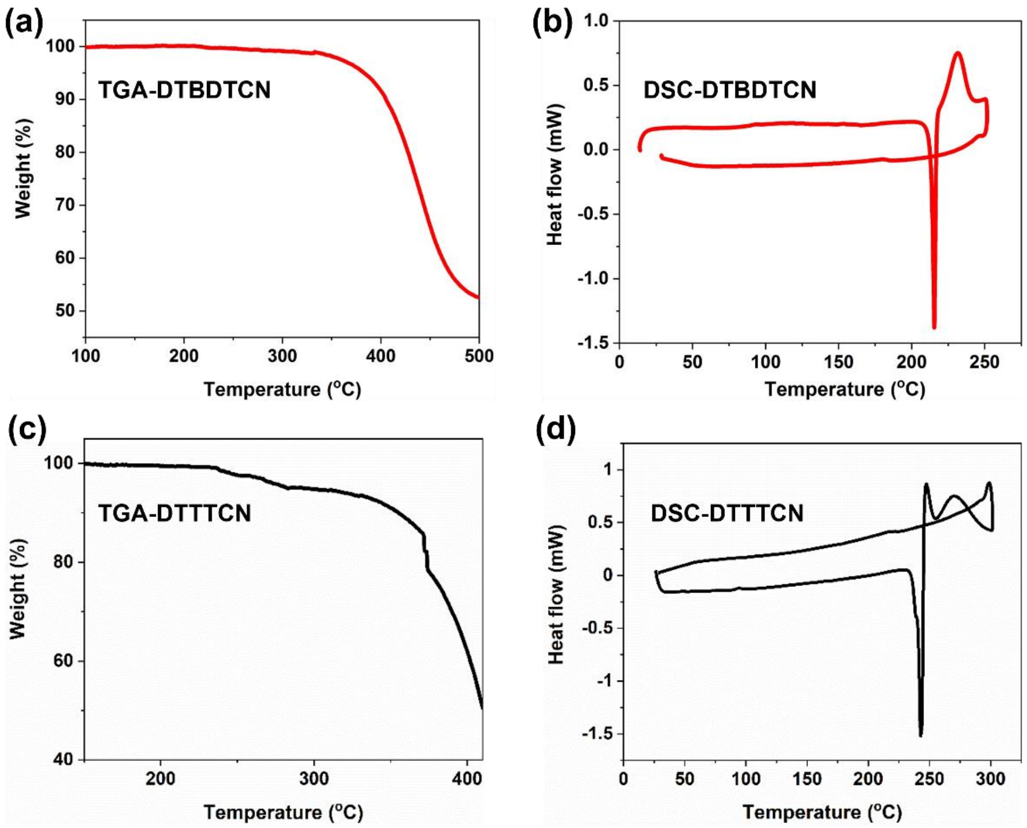

Figure S8. (a) TGA and (b) DSC of **DTBDTCN**; (c) TGA and (d) DSC of **DTTTCN**.

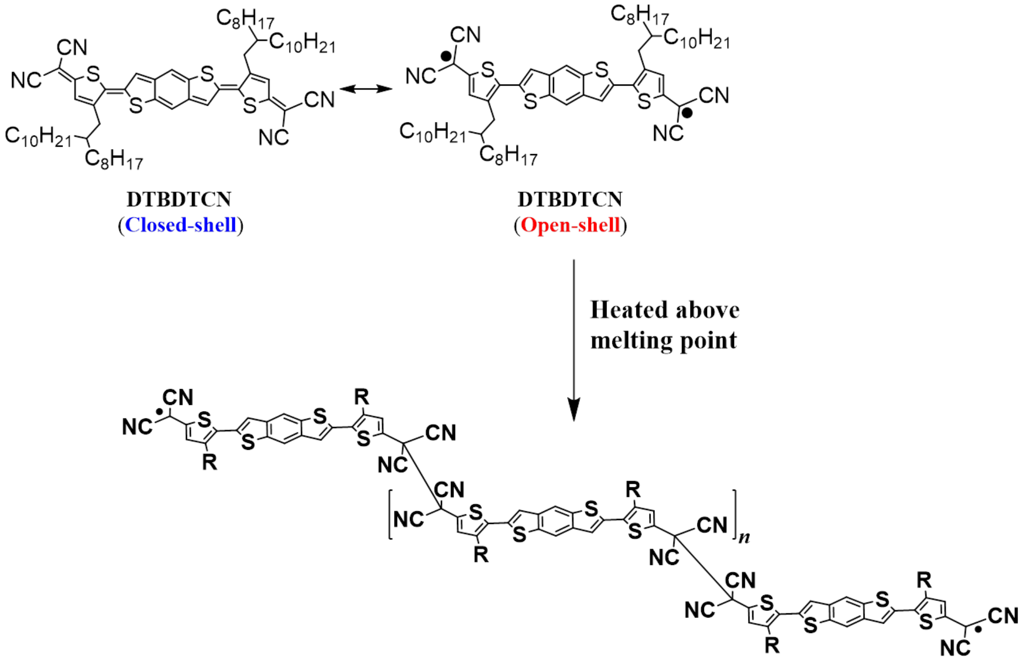

Figure S9. A possible polymerization reaction of the **DTBDTCN** open-shell diradical upon heating above the melting point.

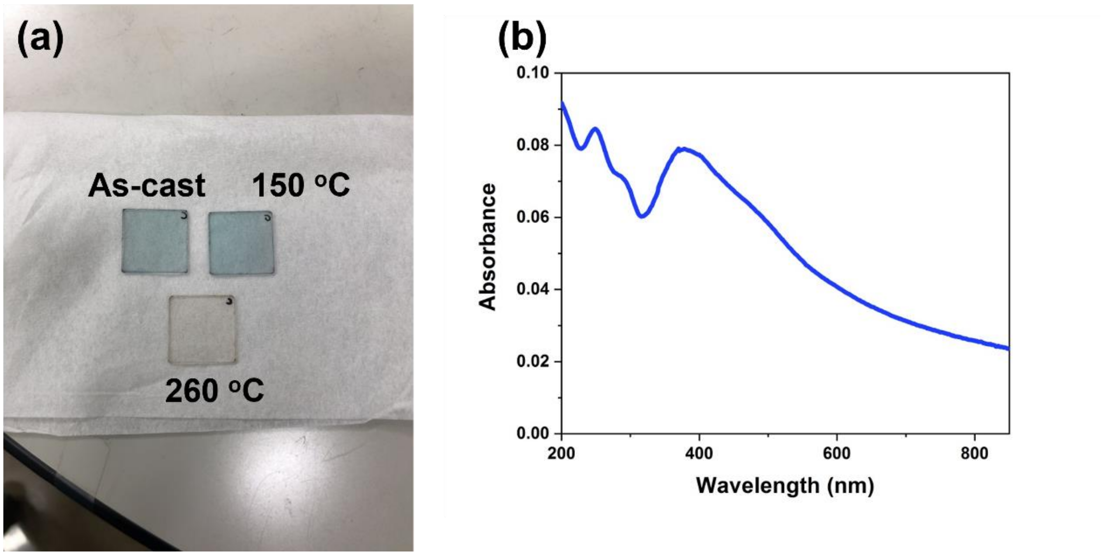

Figure S10. (a) Photograph of the as-cast **DTBDTCN** film, and the **DTBDTCN** films annealed at 150 and 260 °C. (b) Absorption spectrum of the **DTBDTCN** film after annealing at 260 °C.

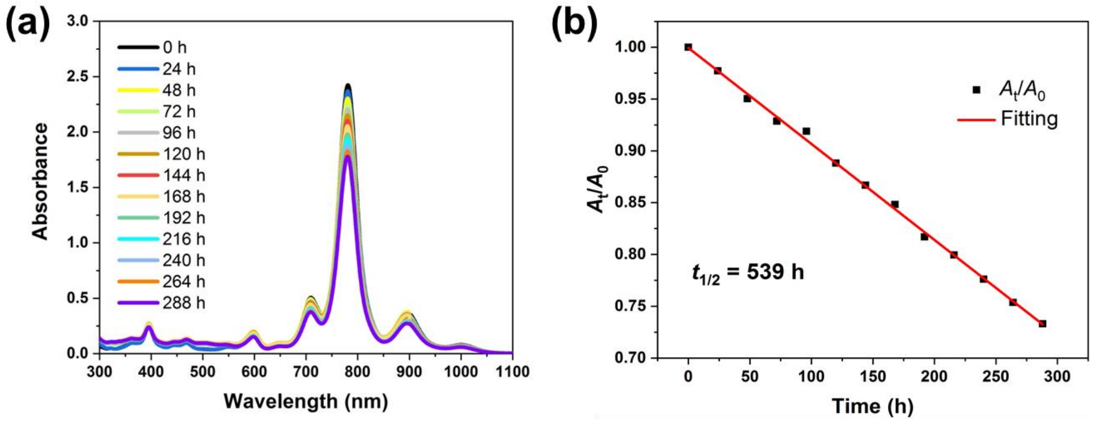

Figure S11. (a) Time-course change in the UV-Vis-NIR absorption spectra of **DTBDTCN** solution in toluene under ambient conditions and (b) plot of A_t/A_0 as a function of time. A_t is the absorbance at 781 nm at time t (h), and A_0 is the initial absorbance at 781 nm.

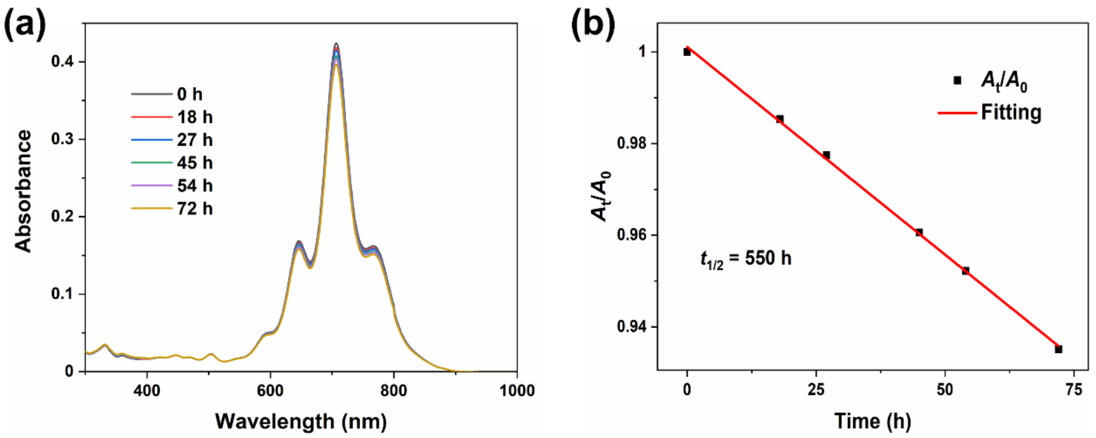

Figure S12. (a) Time-course change in the UV-Vis-NIR absorption spectra of **DTTTCN** solution in toluene under ambient conditions and (b) plot of A_t/A_0 as a function of time. A_t is the absorbance at 707 nm at time t (h), and A_0 is the initial absorbance at 707 nm.

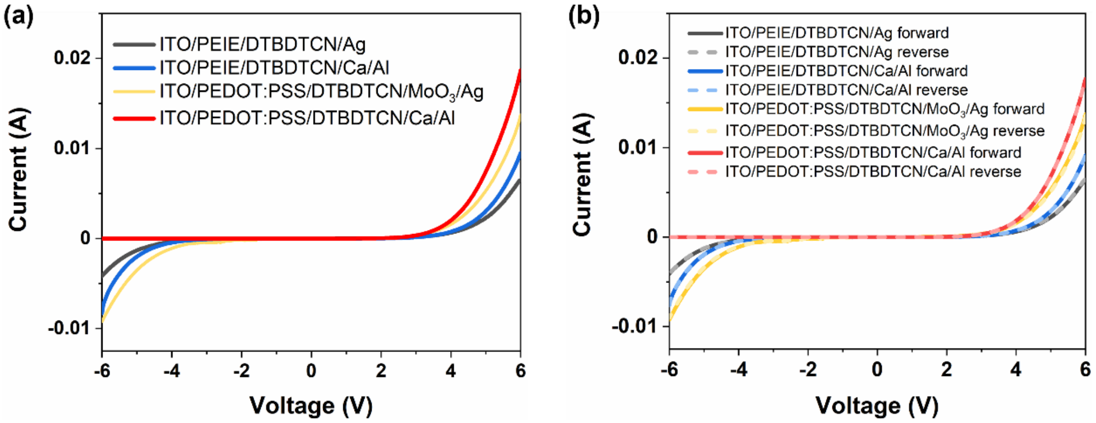

Figure S13. (a) Current-voltage (*I*-*V*) characteristics of different devices. (b) *I*-*V* characteristics of different devices for the forward and reverse sweep of bias voltage with a constant magnetic field of 100 mT.

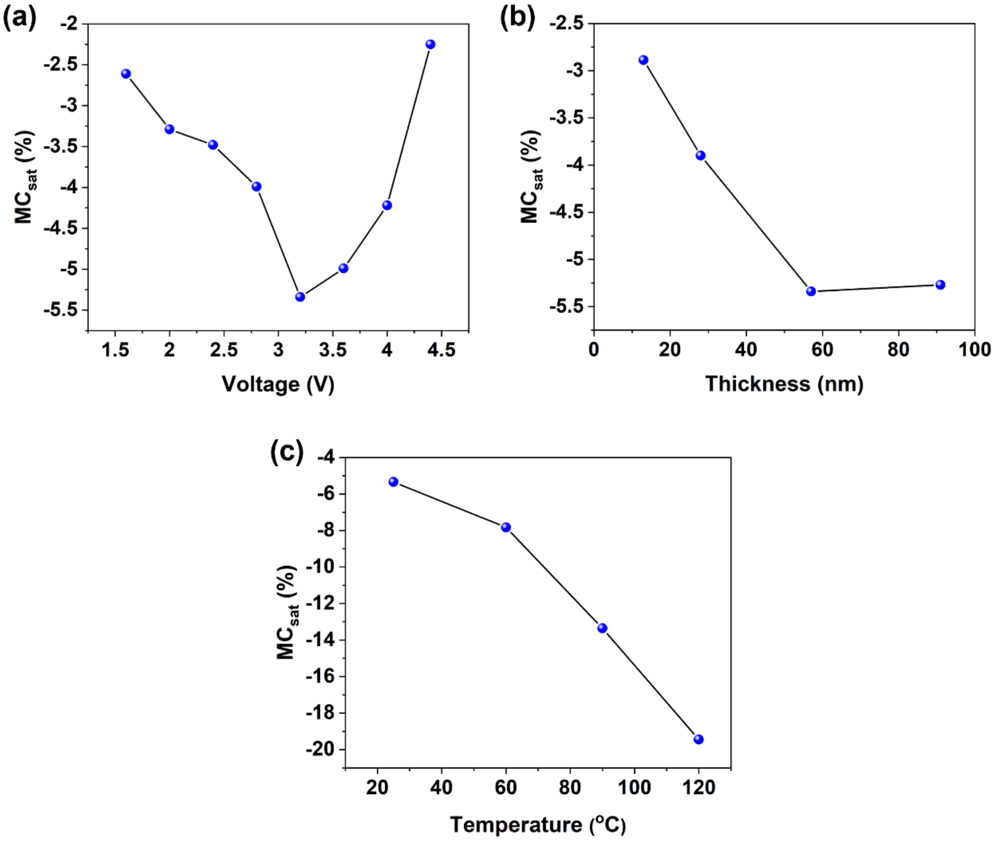

Figure S14. (a) Voltage dependence of MC_{sat} at 25 °C, (b) thickness dependence of MC_{sat} at 25 °C with an applied voltage of 3.2 V, and (c) temperature dependence with an applied voltage of 3.2 V of MC_{sat} for **DTBDTCN** devices.

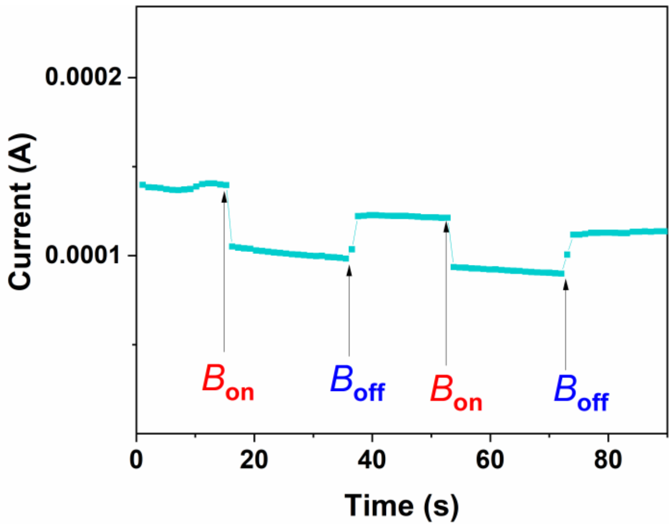

Figure S15. The current vs. time for the **DTBDTCN** diode device with and without the magnetic field of a permanent magnet as shown in **Video S1**. The permanent magnet was moved close to the device at B_{on} and moved away from the device at B_{off}.

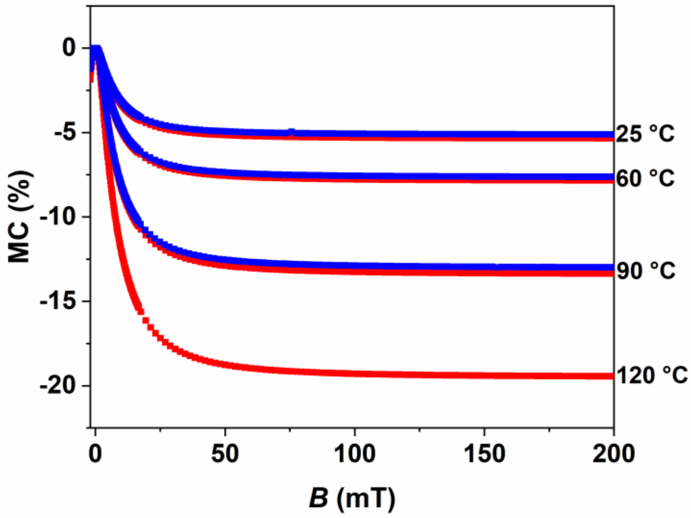

Figure S16. The reversibility of OMAR effect in the temperature range up to 120 °C. Red curves represent MC when the measurement temperature was raised from 25 to 120 °C. Blue curves represent MC when the measurement temperature was lowered from 120 to 25 °C.

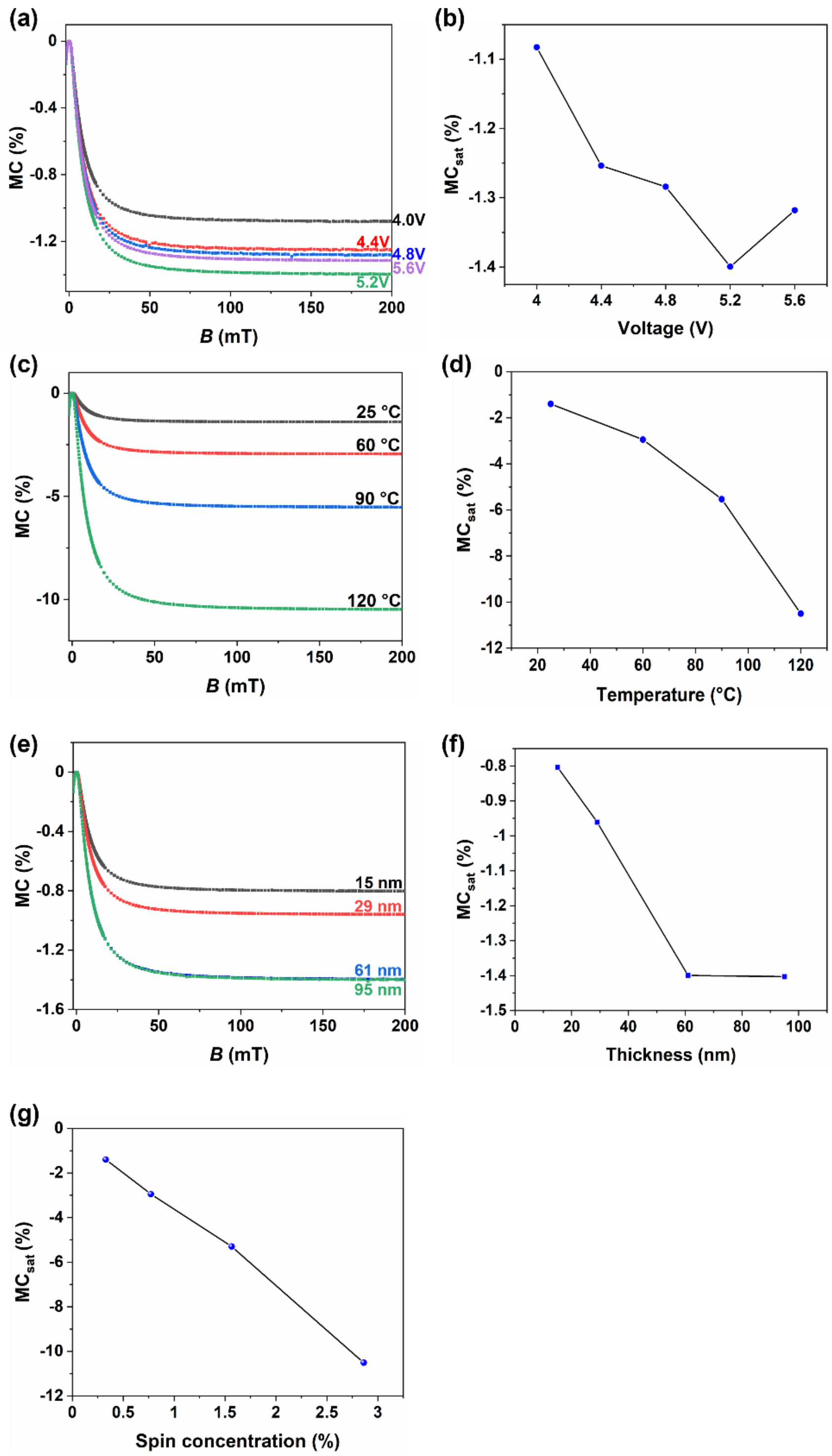

Figure S17. OMAR effect of ITO/PEIE/**DTBDTCN**/Ag. (a) MC vs. *B* with different voltages at 25 °C. (b) Voltage dependence of MC_{sat} at 25 °C. (c) MC vs. *B* at 5.2 V at the different temperatures. (d) Temperature dependence with an applied voltage of 5.2 V of MC_{sat}. (e) MC vs. *B* at 5.2 V with different **DTBDTCN** film thicknesses. (f) Thickness dependence of MC_{sat} at 25 °C with an applied voltage of 5.2 V. (g) MC_{sat} as a function of the spin concentration in **DTBDTCN** determined by ESR.

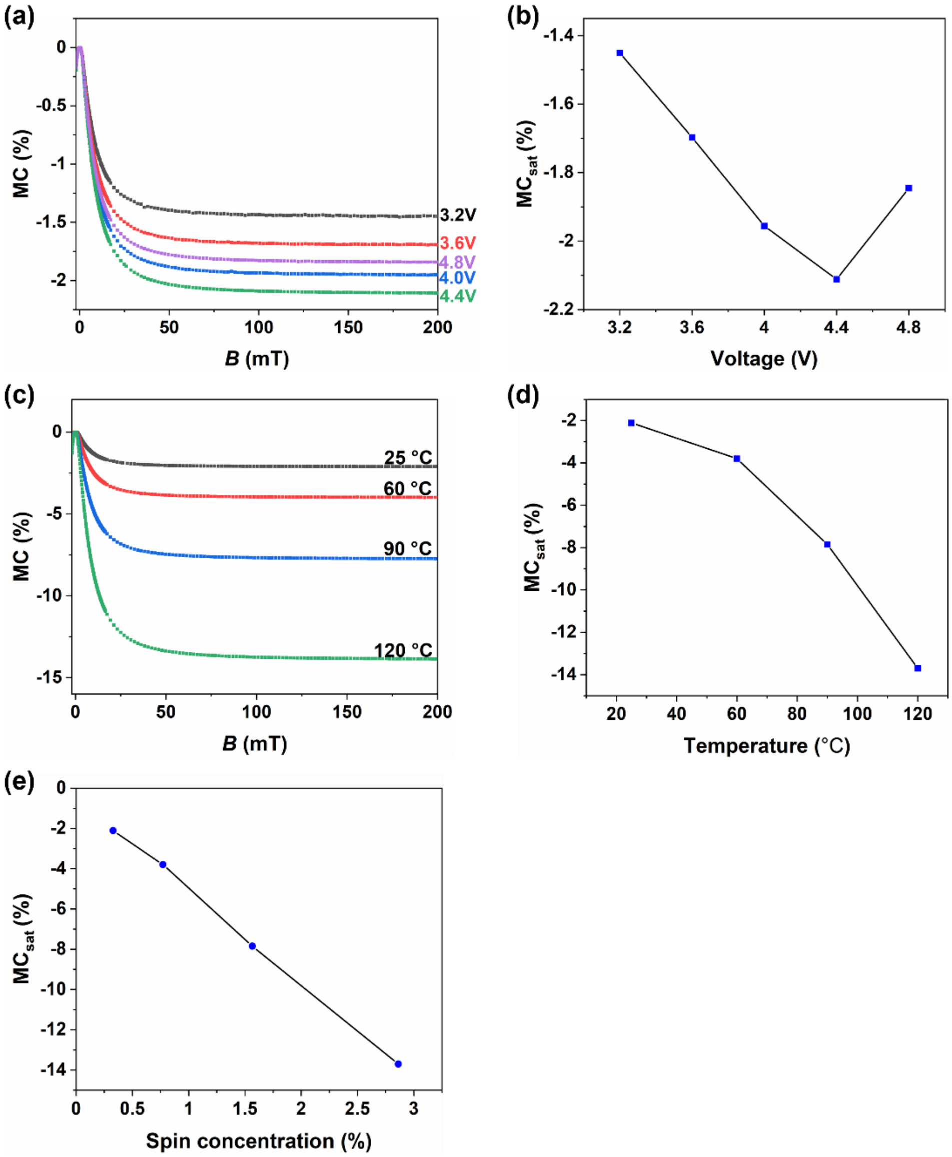

Figure S18. OMAR effect of ITO/PEIE/**DTBDTCN**/Ca/Al. (a) MC vs. *B* with different voltages at 25 °C. (b) Voltage dependence of MC_{sat} at 25 °C. (c) MC vs. *B* at 4.4 V at the different temperatures. (d) Temperature dependence with an applied voltage of 4.4 V of MC_{sat}. (e) MC_{sat} as a function of the spin concentration in **DTBDTCN** determined by ESR.

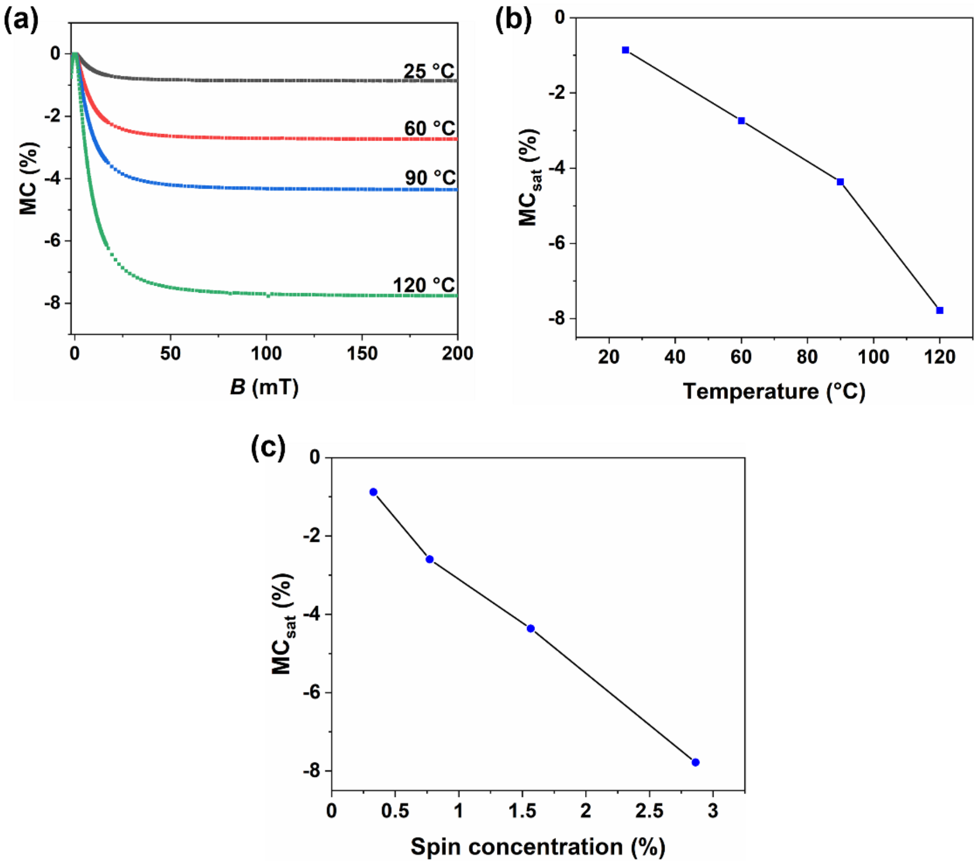

Figure S19. OMAR effect of ITO/PEIE/**DTBDTCN**/Ag at −5.2V. (a) MC vs. B at −5.2 V at the different temperatures. (b) Temperature dependence with an applied voltage of −5.2 V of MC_{sat}. (c) MC_{sat} as a function of the spin concentration in DTBDTCN determined by ESR.

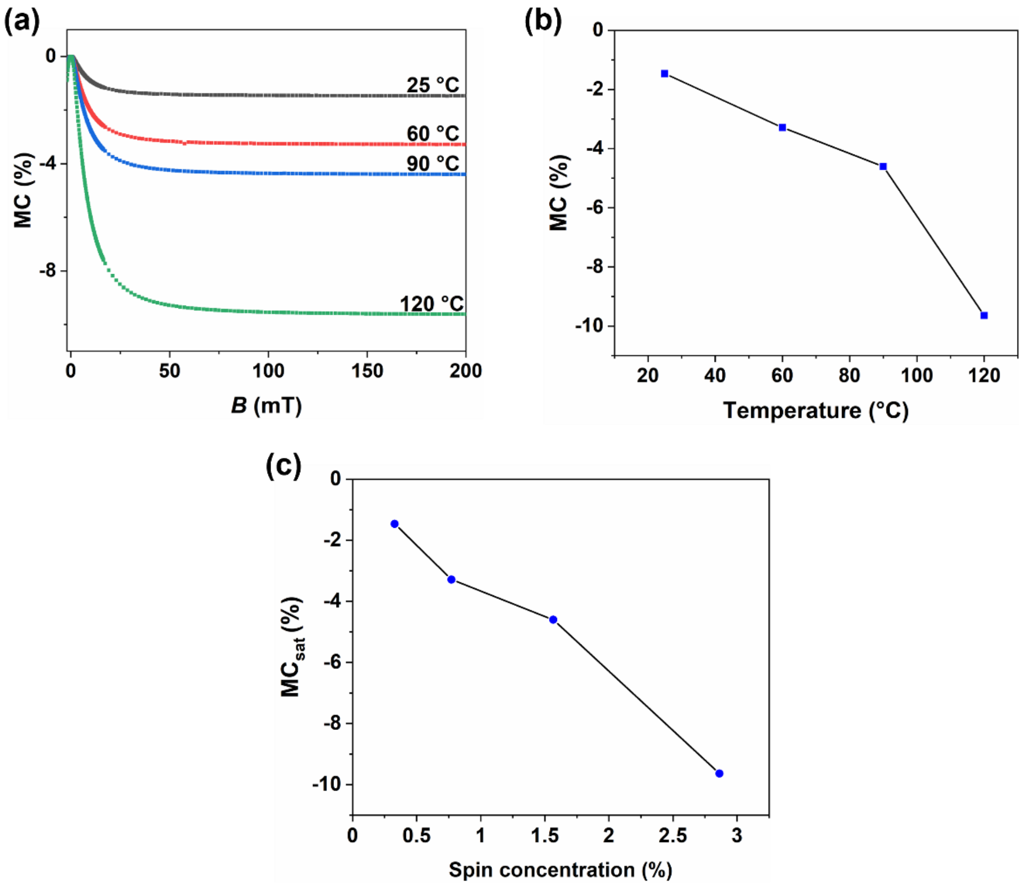

Figure S20. OMAR effect of ITO/PEIE/**DTBDTCN**/Ca/Al at −4.4V. (a) MC vs. *B* at −4.4 V at different temperatures. (b) Temperature dependence with an applied voltage of −4.4 V of MC_{sat}. (c) MC_{sat} as a function of the spin concentration in **DTBDTCN** determined by ESR.

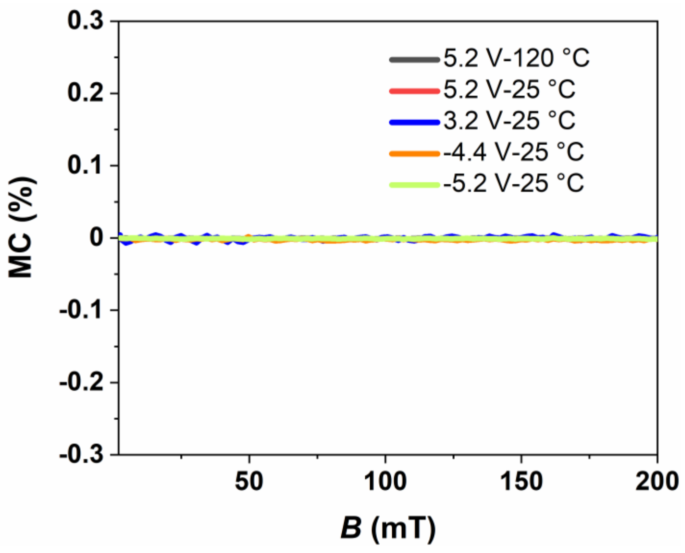

Figure S21. MC of ITO/PEDOT:PSS/**DTBDTCN/**MoO_3/Ag device measured under different conditions: −5.2 V to 5.2 V at 25 °C and 5.2 V at 120 °C.

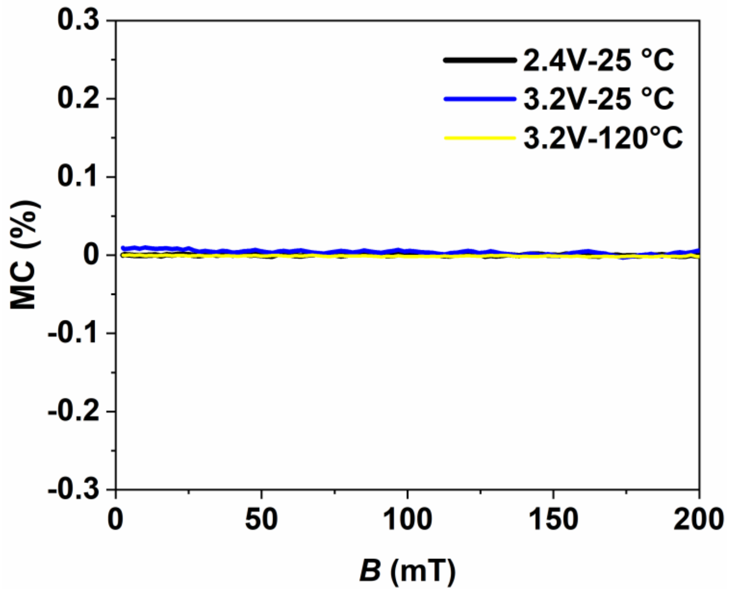

Figure S22. MC of **DTTTCN** device measured under different conditions: 2.4 V at 25 °C, 3.2V at 25 °C, and 3.2V at 120 °C.

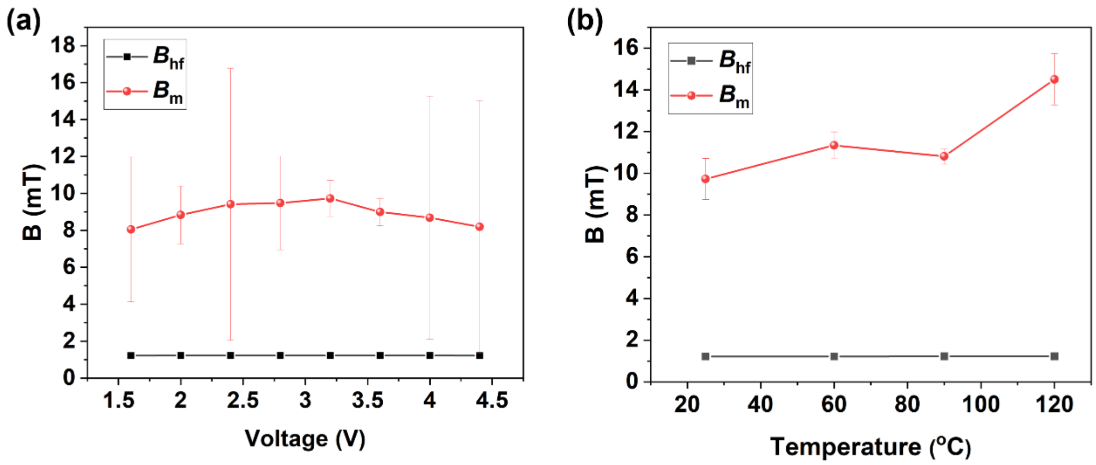

Figure S23. (a) Voltage and (b) temperature dependence of B_{hf} and B_m in **DTBDTCN** devices obtained by fitting the MC curves. The voltage for (b) is 3.2V.

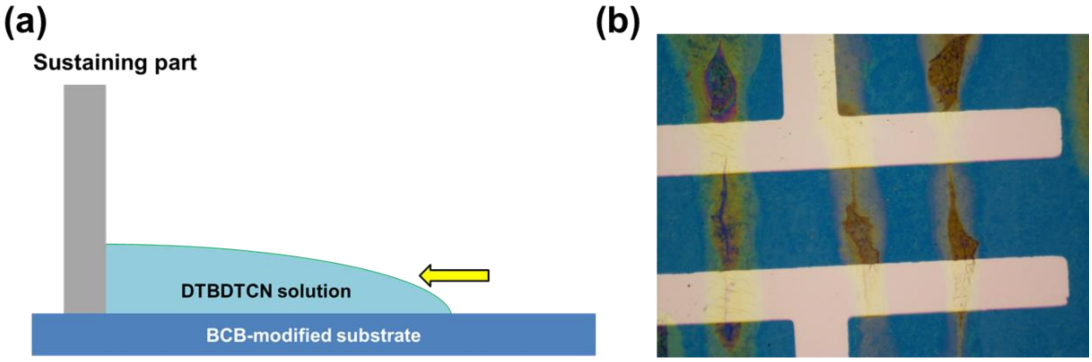

Figure S24. (a) Schematic of the edge-casting method for **DTBDTCN** thin film preparation. A 100 μL droplet of a 0.5 mg mL^{-1} solution of **DTBDTCN** in a mixed solvent of toluene and chlorobenzene (3:1 in v/v) is placed at the edge of a sustaining part on a BCB-modified SiO_2/Si substrate. The domains grow along the direction of the solvent evaporation (yellow arrow). After solvent evaporation, the film is thermally annealed at 150 °C for 30 min. (b) Optical microscope image of the OFET device based on an edge-cast film of **DTBDTCN**. The growth direction of the domains is parallel to the OFET channel.

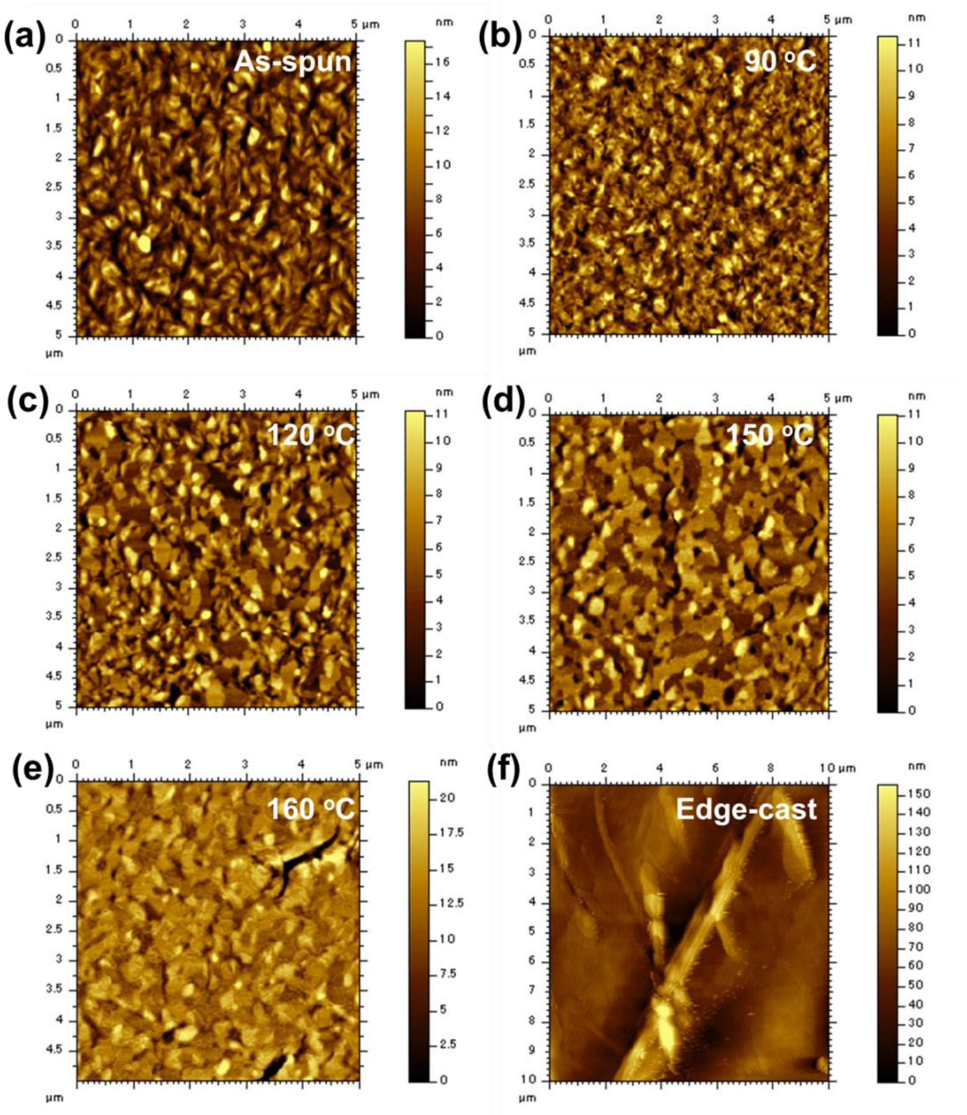

Figure S25. AFM images of (a) the as-spun **DTBDTCN** film, spin-coated **DTBDTCN** films after annealing at (b) 90 °C, (c) 120 °C, (d) 150 °C, and (e) 160 °C, and (f) the edge-cast **DTBDTCN** film.

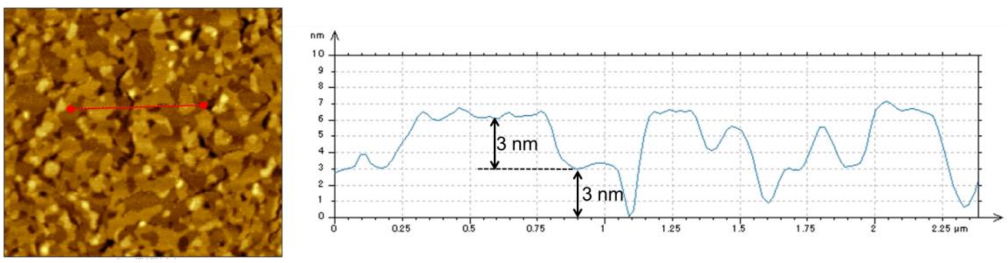

Figure S26. Line profile of the height image for the spin-coated **DTBDTCN** film annealed at 150 °C.

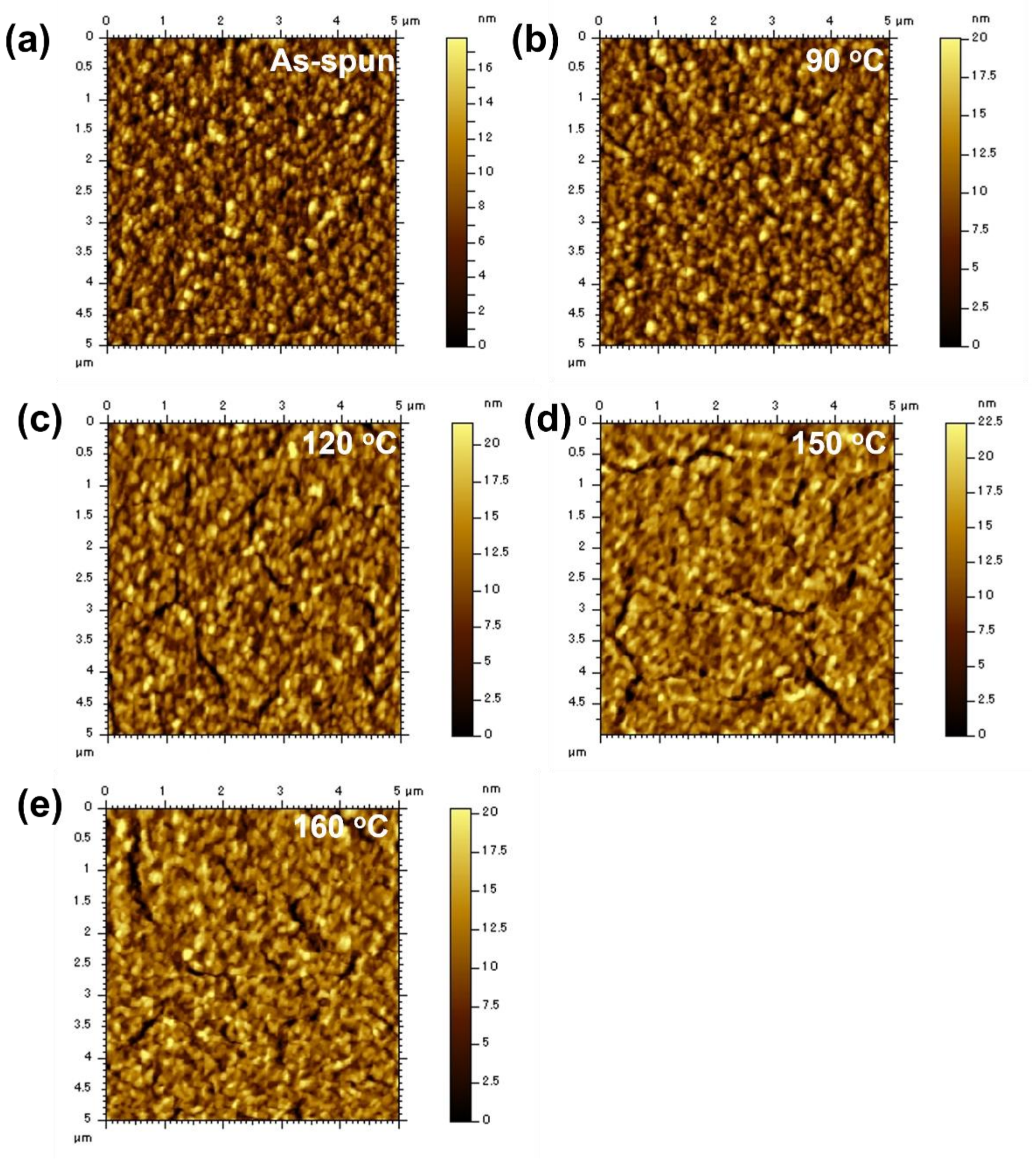

Figure S27. AFM images of (a) the as-spun **DTTTCN** film, spin-coated **DTTTCN** films after annealing at (b) 90 °C, (c) 120 °C, (d) 150 °C, and (e) 160 °C.

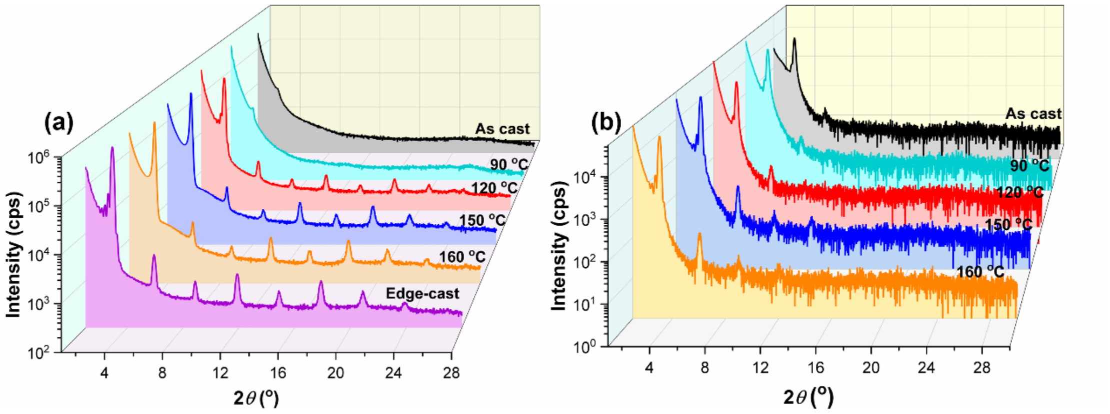

Figure S28. Out-of-plane XRD patterns of the thin films of (a) **DTBDTCN** and (b) **DTTTCN**.

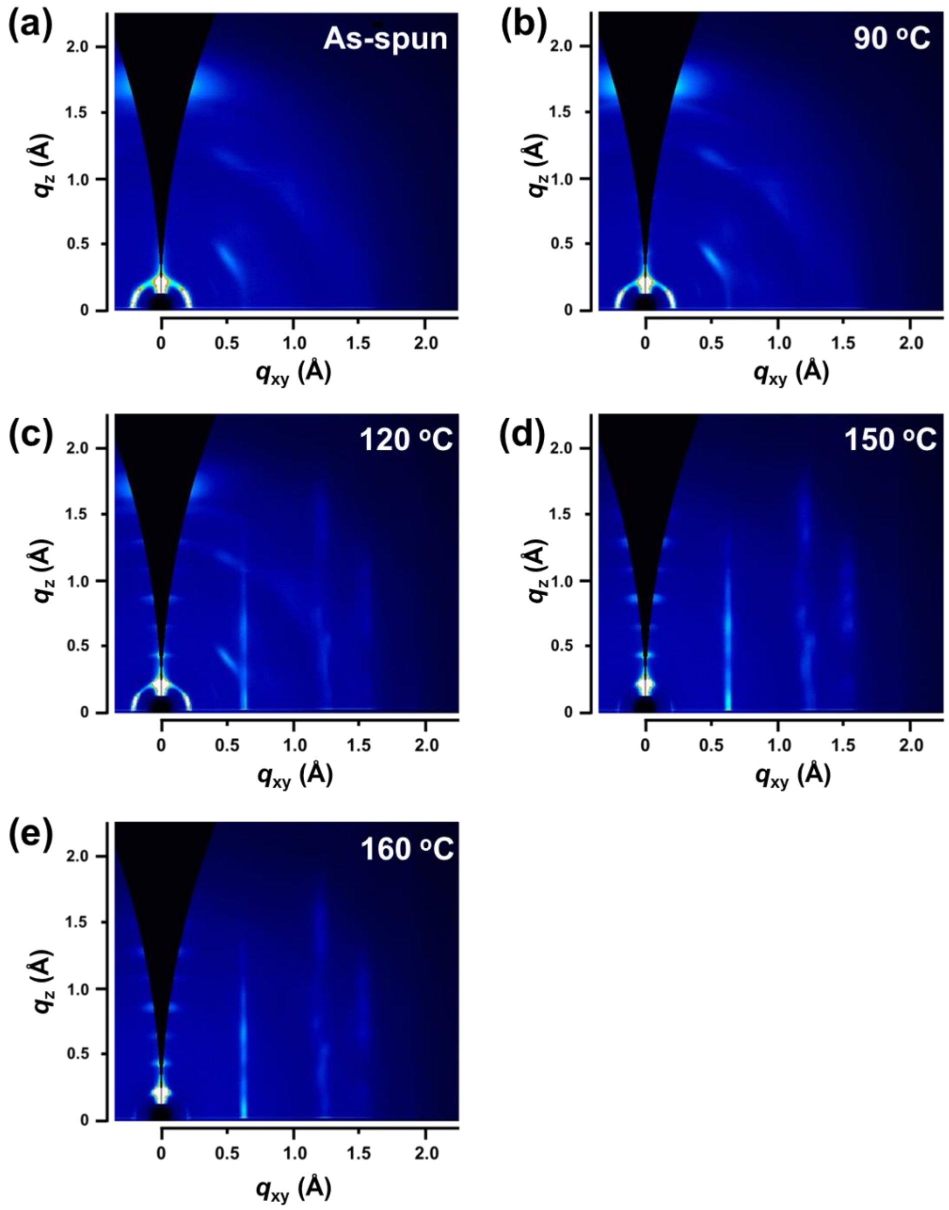

Figure S29. GIWAXS patterns of the (a) as-spun **DTBDTCN** film and spin-coated **DTBDTCN** films after annealing at (b) 90 °C, (c) 120 °C, (d) 150 °C, and (e) 160 °C.

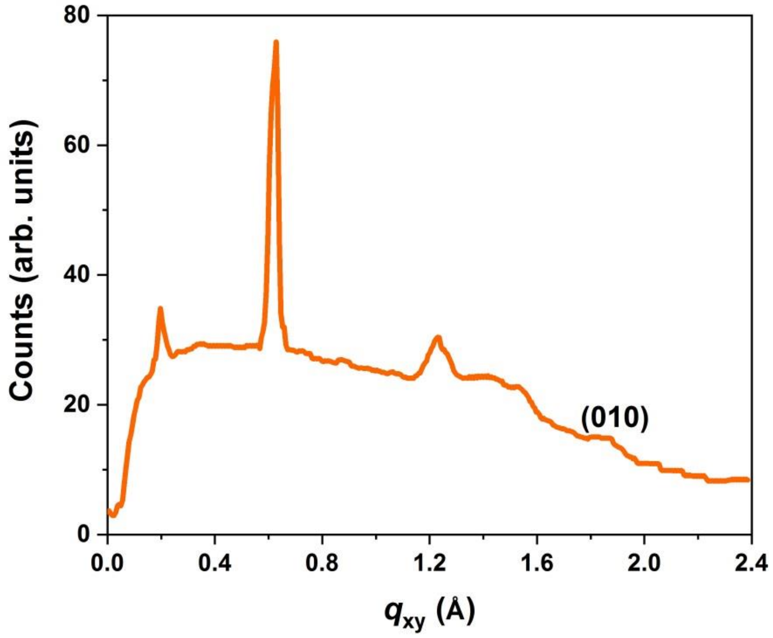

Figure S30. Line profile along the q_{xy} axis (in-plane) of the GIWAXS image of **DTBDTCN** in the spin-coated film after annealing at 150 °C.

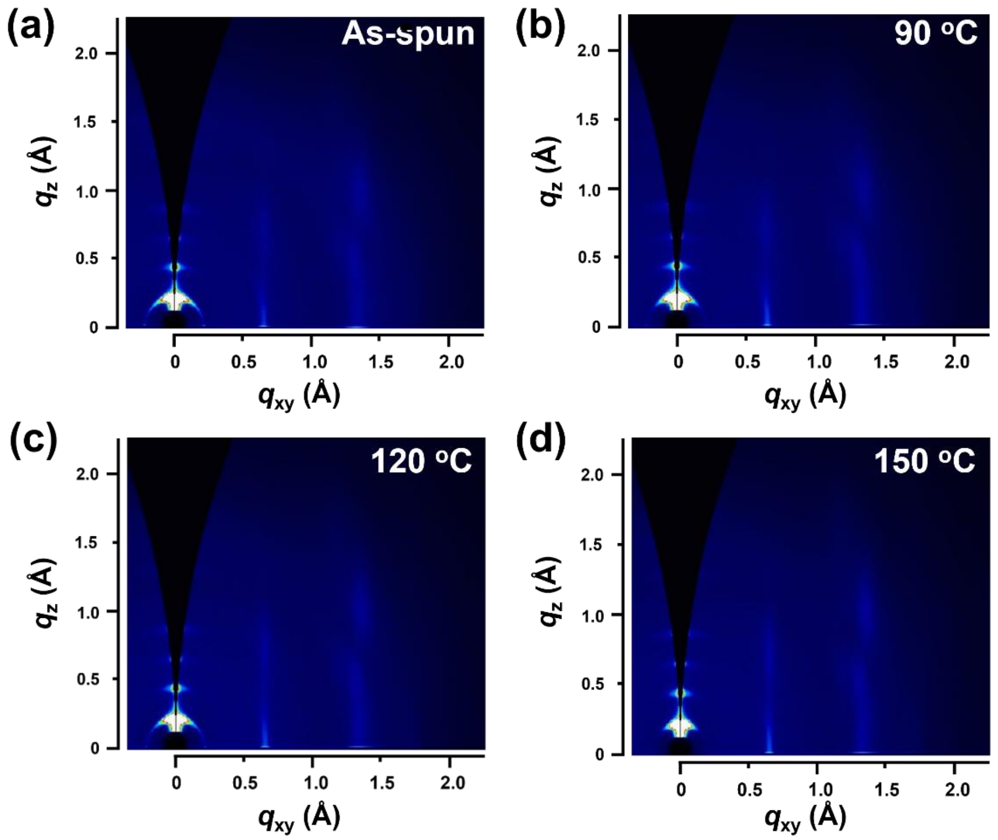

Figure S31. GIWAXS patterns of the (a) as-spun **DTTTCN** film and spin-coated **DTTTCN** films after annealing at (b) 90 °C, (c) 120 °C, (d) 150 °C, and (e) 160 °C.

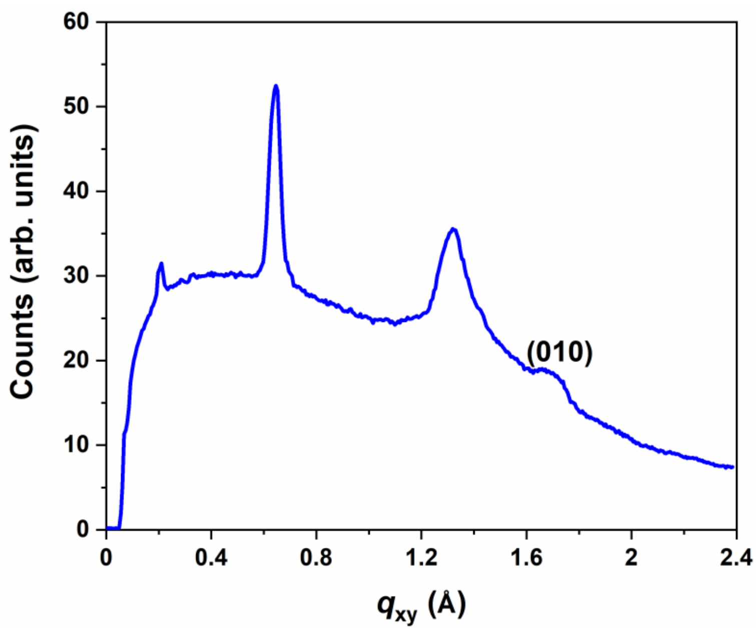

Figure S32. Line profile along the q_{xy} axis (in-plane) of the GIWAXS image of **DTTTCN** in the spin-coated film after annealing at 150 °C.

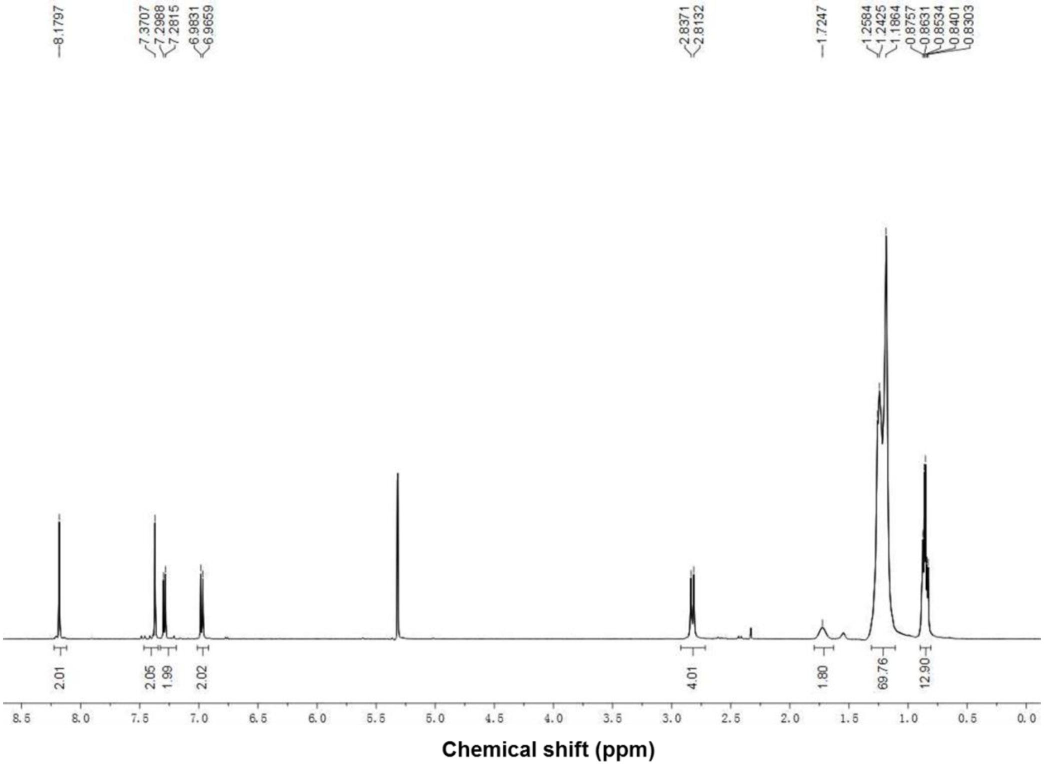

Figure S33. ^{1}H NMR spectrum of 2,6-bis(3-(2-octyldodecyl)thiophen-2-yl)benzo[1,2-b:4,5-b′] dithiophene (**3**) in CD_2Cl_2.

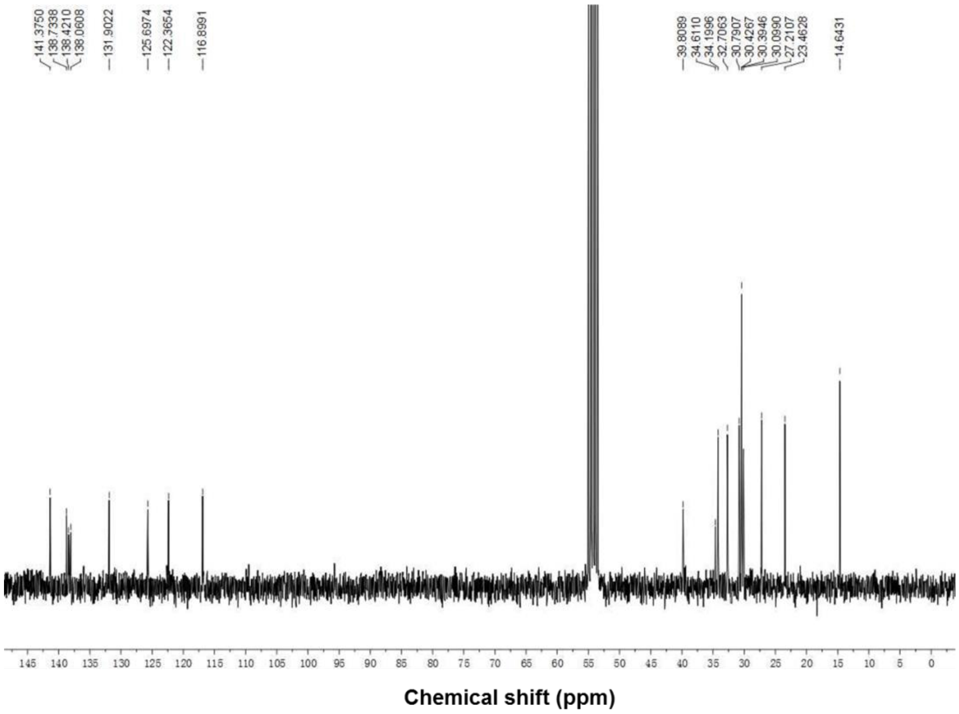

Figure S34. ^{13}C NMR spectrum of 2,6-bis(3-(2-octyldodecyl)thiophen-2-yl)benzo[1,2-b:4,5-b′] dithiophene (**3**) in CD_2Cl_2.

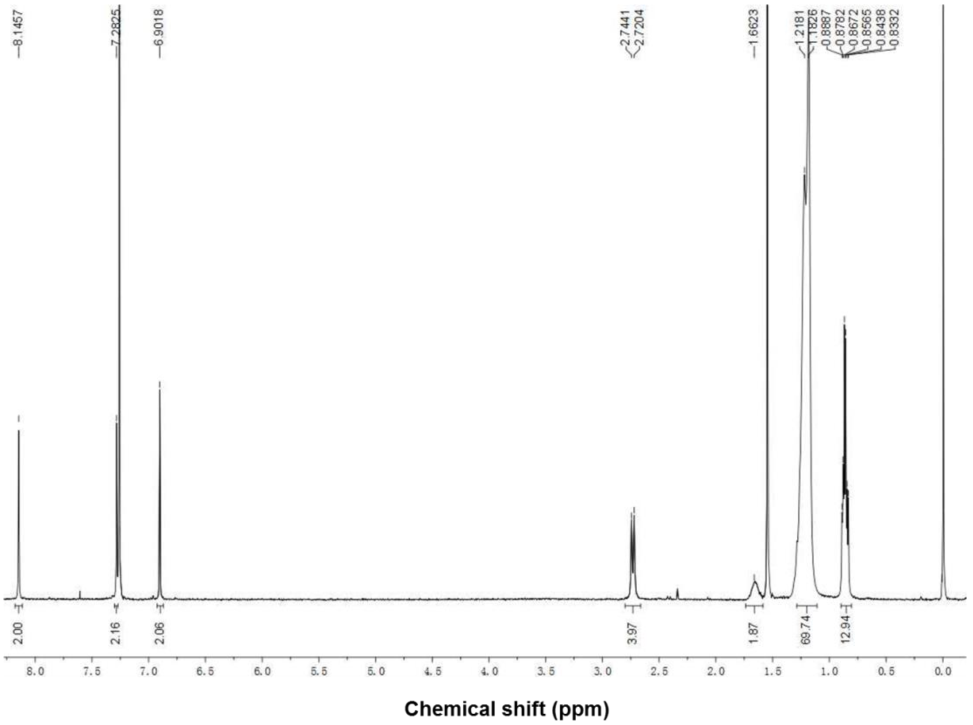

Figure S35. ^{1}H NMR spectrum of 2,6-bis(5-bromo-3-(2-octyldodecyl)thiophen-2-yl)benzo [1,2-b:4,5-b′]dithiophene (**4**) in $CDCl_3$.

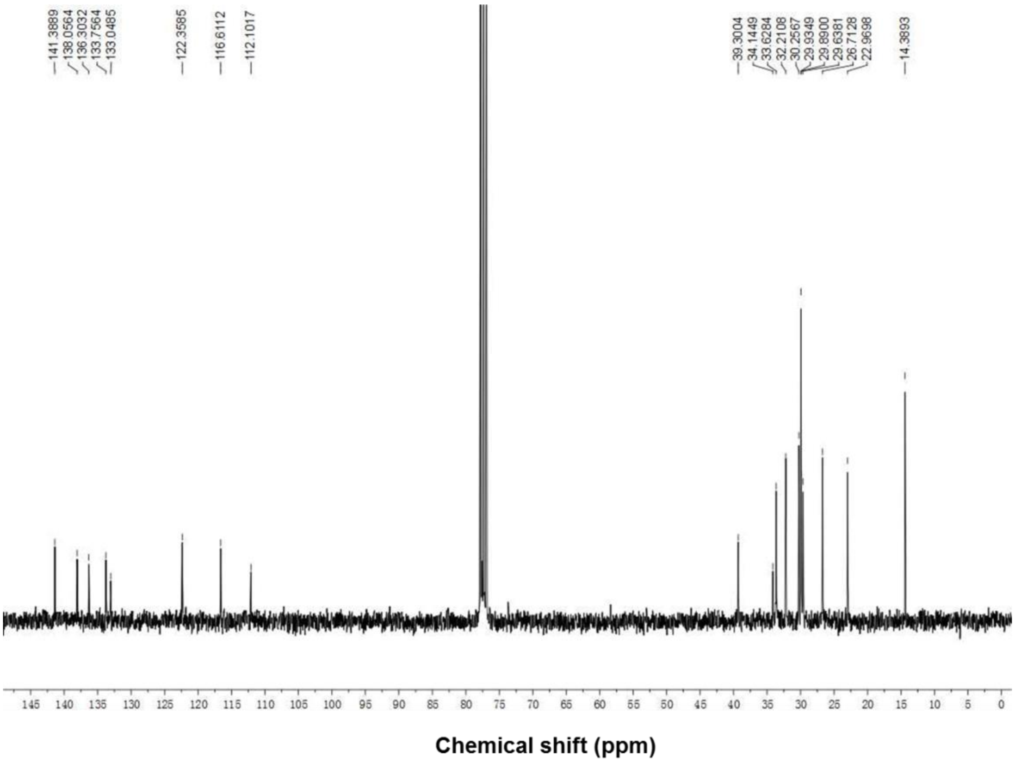

Figure S36. ^{13}C NMR spectrum of 2,6-bis(5-bromo-3-(2-octyldodecyl)thiophen-2-yl)benzo [1,2-b:4,5-b′]dithiophene (**4**) in $CDCl_3$.

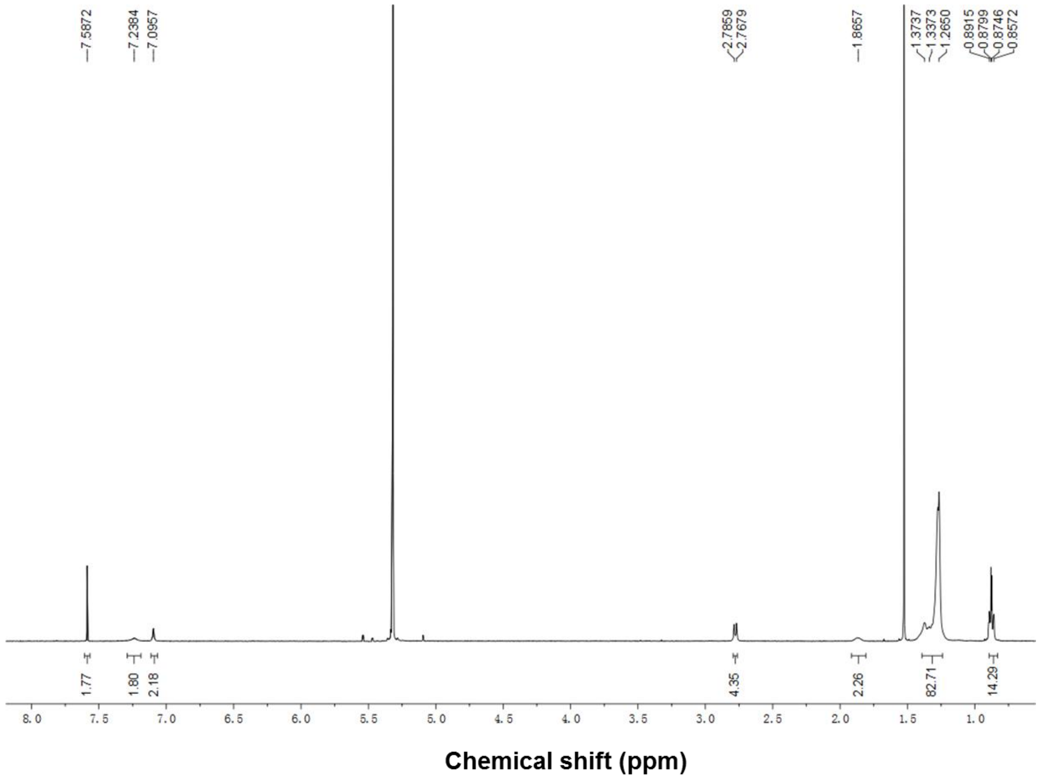

Figure S37. ^{1}H NMR spectrum of **DTBDTCN** in CD_2Cl_2 at −60 °C.

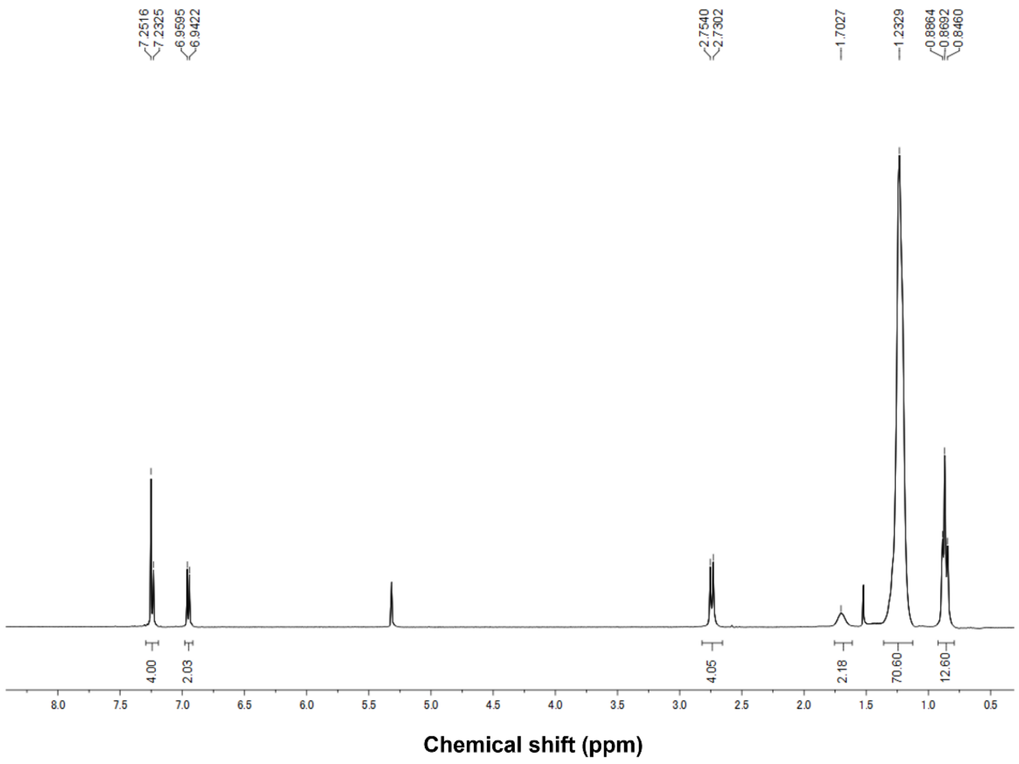

Figure S38. ^{1}H NMR spectrum of 2,5-bis(3-(2-octyldodecyl)thiophen-2-yl)thieno[3,2-b] thiophene (**6**) in CD_2Cl_2.

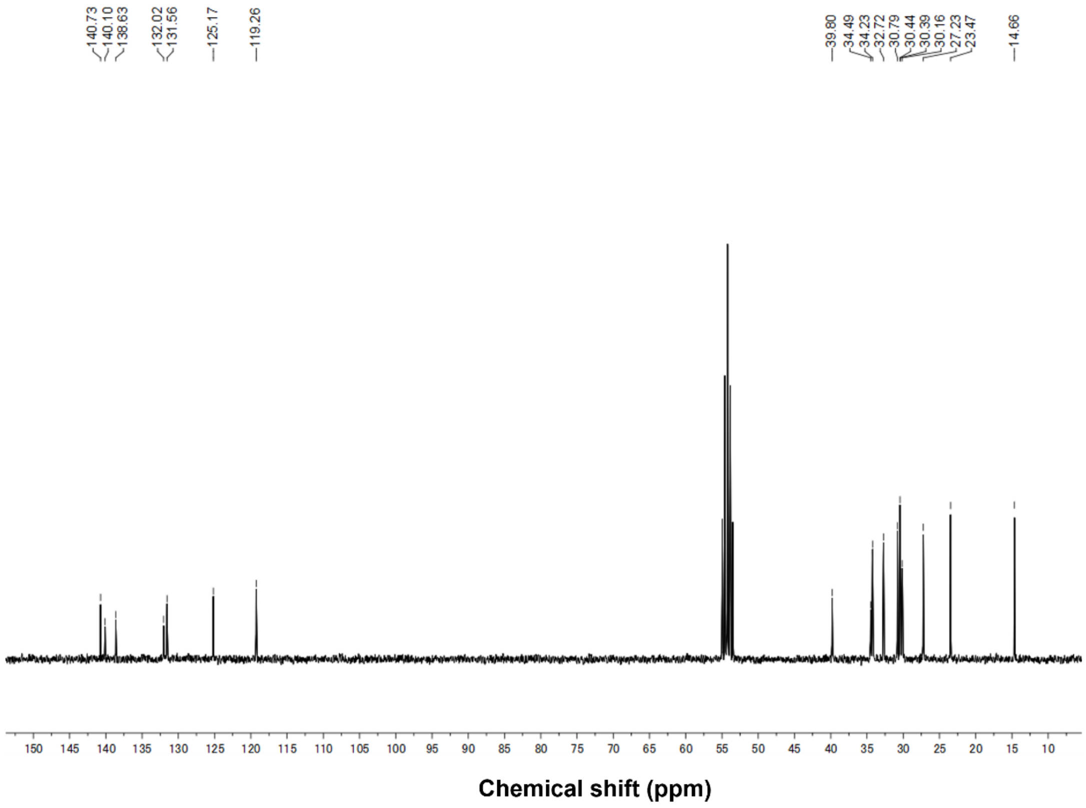

Figure S39. ^{13}C NMR spectrum of 2,5-bis(3-(2-octyldodecyl)thiophen-2-yl)thieno[3,2-b] thiophene (**6**) CD_2Cl_2.

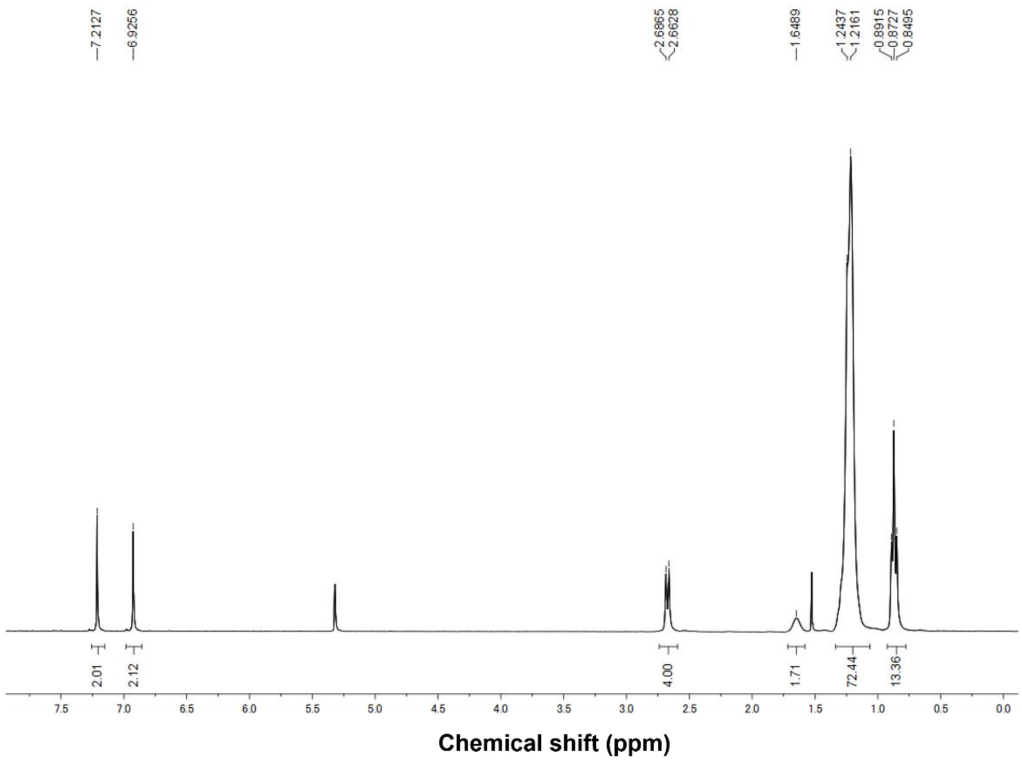

Figure S40. ^{1}H NMR spectrum of 2,5-bis(5-bromo-3-(2-octyldodecyl)thiophen-2-yl)thieno [3,2-b]thiophene (**7**) CD_2Cl_2.

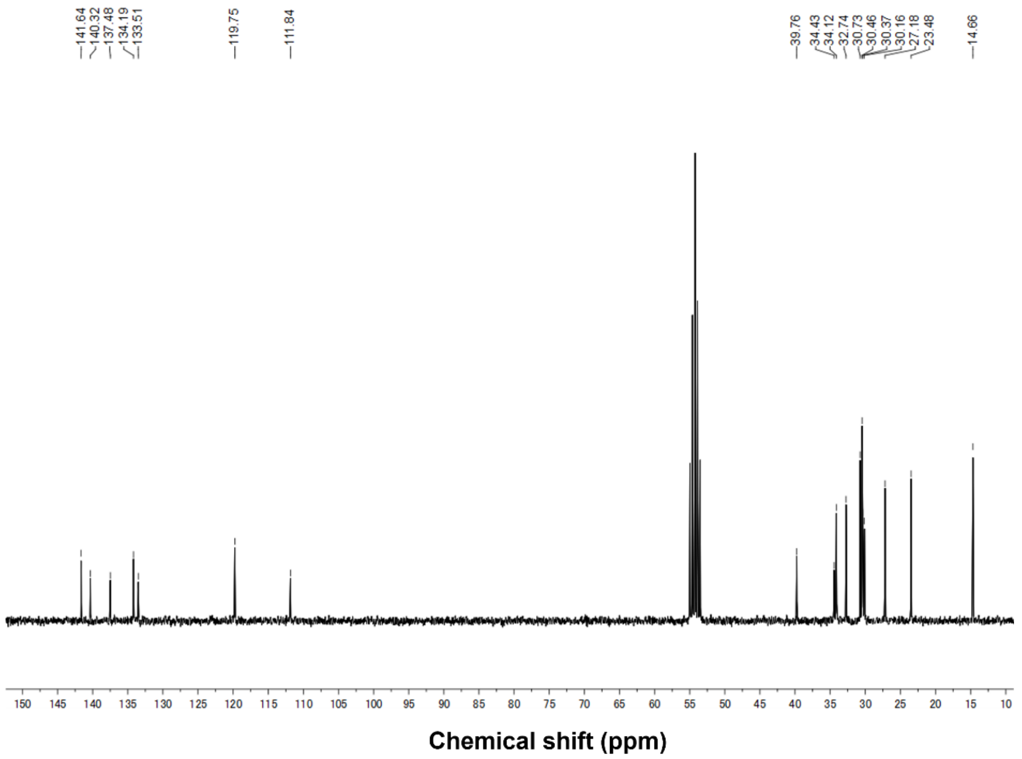

Figure S41. ^{13}C NMR spectrum of 2,5-bis(5-bromo-3-(2-octyldodecyl)thiophen-2-yl)thieno [3,2-b]thiophene (**7**) CD_2Cl_2.

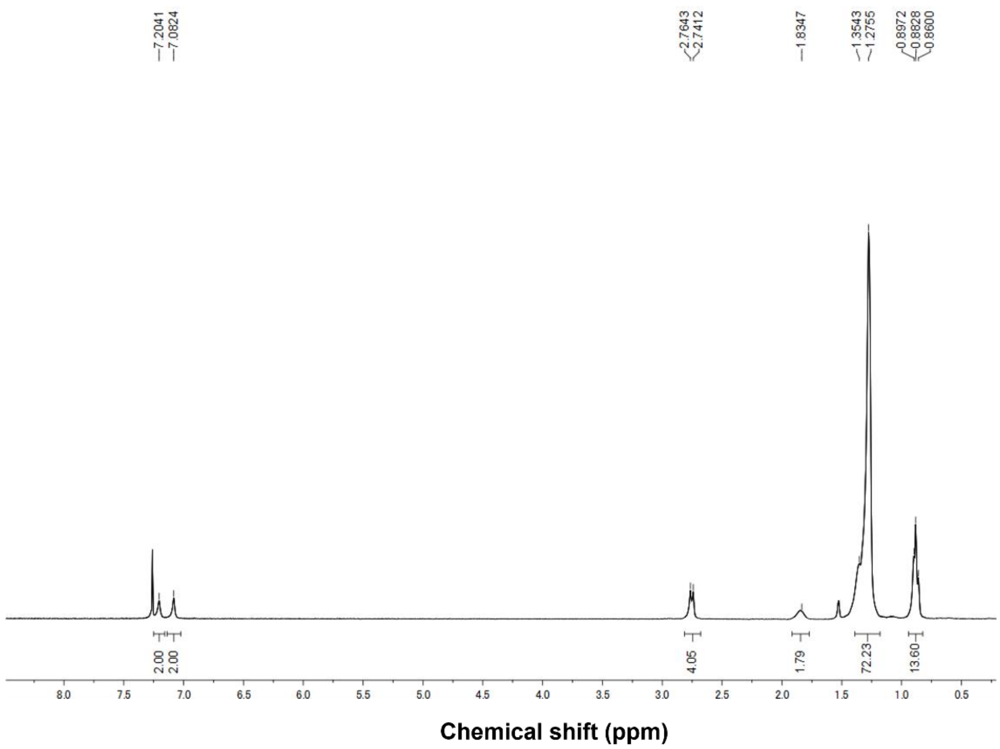

Figure S42. ^{1}H NMR spectrum of **DTTTCN** in $CDCl_3$ at 25 °C.

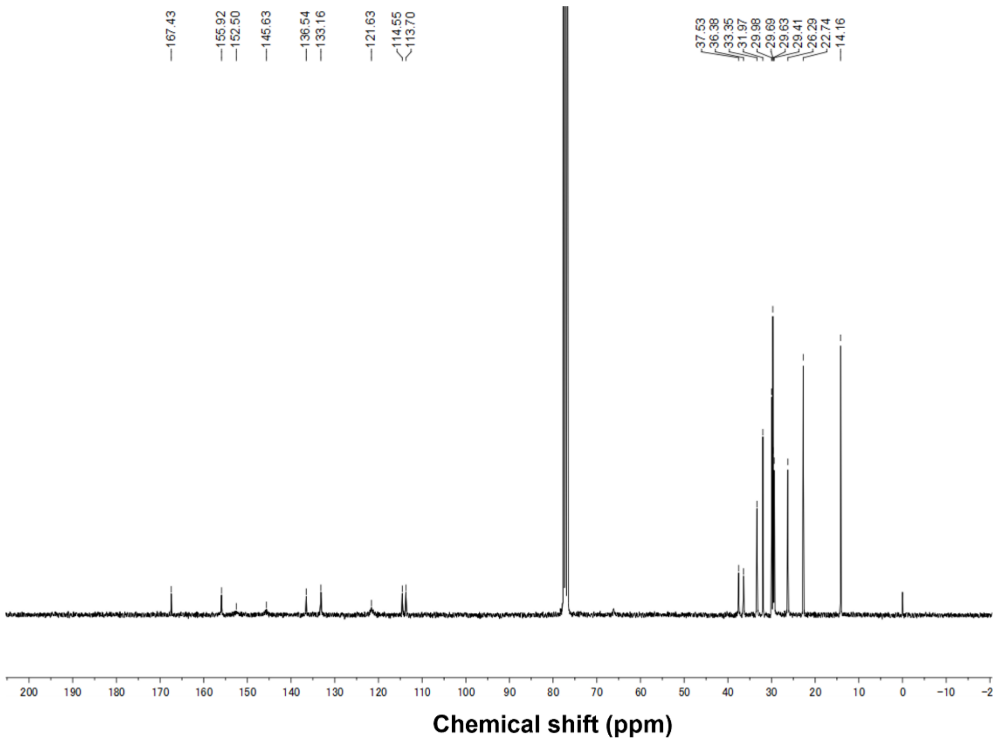

Figure S43. ^{13}C NMR spectrum of **DTTTCN** in $CDCl_3$ at 25 °C.

Reference

[1] C. Wang, H. Dong, W. Hu, Y. Liu, D. Zhu, *Chem. Rev.* **2012**, *112*, 2208–2267.

[2] A. Shimizu, M. Uruichi, K. Yakushi, H. Matsuzaki, H. Okamoto, M. Nakano, Y. Hirao, K. Matsumoto, H. Kurata, T. Kubo, *Angew. Chem. Int. Ed.* **2009**, *48*, 5482−5486.

[3] Z. Sun, K.-W. Huang, J. Wu, *J. Am. Chem. Soc.* **2011**, *133*, 11896–11899.

[4] X. Zhu, H. Tsuji, K. Nakabayashi, S. Ohkoshi, E. Nakamura, *J. Am. Chem. Soc.* **2011**, *133*, 16342–16345.

[5] C. Zhang, S. M. Rivero, W. Liu, D. Casanova, X. Zhu, J. Casado, *Angew. Chem. Int. Ed.* **2019**, *58*, 11291–11295.

[6] K. Yang, X. Zhang, A. Harbuzaru, L. Wang, Y. Wang, C. Koh, H. Guo, Y. Shi, J. Chen, H. Sun, K. Feng, M. C. Ruiz Delgado, H. Y. Woo, R. P. Ortiz, X. Guo, *J. Am. Chem. Soc.* **2020**, *142*, 4329−4340.
